# Persister control by leveraging dormancy associated reduction of antibiotic efflux

**DOI:** 10.1371/journal.ppat.1010144

**Published:** 2021-12-10

**Authors:** Sweta Roy, Ali Adem Bahar, Huan Gu, Shikha Nangia, Karin Sauer, Dacheng Ren

**Affiliations:** 1 Department of Biomedical and Chemical Engineering, Syracuse University, Syracuse, New York, United States of America; 2 Department of Biological Sciences, Binghamton University, Binghamton, New York, United States of America; 3 Department of Civil and Environmental Engineering, Syracuse University, Syracuse, New York, United States of America; 4 Department of Biology, Syracuse University, Syracuse, New York, United States of America; "INSERM", FRANCE

## Abstract

Persistent bacterial infections do not respond to current antibiotic treatments and thus present a great medical challenge. These conditions have been linked to the formation of dormant subpopulations of bacteria, known as persister cells, that are growth-arrested and highly tolerant to conventional antibiotics. Here, we report a new strategy of persister control and demonstrate that minocycline, an amphiphilic antibiotic that does not require active transport to penetrate bacterial membranes, is effective in killing *Escherichia coli* persister cells [by 70.8 ± 5.9% (0.53 log) at 100 μg/mL], while being ineffective in killing normal cells. Further mechanistic studies revealed that persister cells have reduced drug efflux and accumulate more minocycline than normal cells, leading to effective killing of this dormant subpopulation upon wake-up. Consistently, eravacycline, which also targets the ribosome but has a stronger binding affinity than minocycline, kills persister cells by 3 logs when treated at 100 μg/mL. In summary, the findings of this study reveal that while dormancy is a well-known cause of antibiotic tolerance, it also provides an Achilles’ heel for controlling persister cells by leveraging dormancy associated reduction of drug efflux.

## Author summary

Bacterial persister cells are dormant phenotypic variants that are highly tolerant to most antibiotics; and thus, present a major challenge to infection control. This motivated us to develop new strategies that can specifically target the persister population. It is known that persister formation is associated with reduced membrane potential and cellular activities. Thus, we hypothesize that persister cells have reduced drug efflux compared to normal cells and accumulate more antimicrobial agents that can penetrate the membranes of persister cells. By testing this hypothesis, we developed a new set of criteria for selecting persister control agents and demonstrated effective control of *Escherichia coli* persister cells by minocycline, rifamycin SV, and eravacycline. Our results revealed that these agents are more effective against persister cells than normal cells and the killing occurred during persister wake-up. Collectively, these results demonstrate a new strategy for persister control by leveraging dormancy associated changes in bacterial physiology. The findings may contribute to future drug discovery and the treatment of persistent infections.

## Introduction

Despite the past decades of success in infection control by antibiotics, persistent bacterial infections remain challenging such as tuberculosis [1], Lyme disease [2], and chronical infections associated with cystic fibrosis [3] and implanted medical devices [4]. These seemingly different disease conditions face the same challenge, bacterial dormancy, which leads to extremely high levels of antibiotic tolerance. An important mechanism of dormancy is the formation of persister cells, a small subpopulation of dormant phenotypic variants that are highly tolerant to different stresses including antibiotics [5–7]. There are increasing evidences of persistence in clinical settings, such as chronic infections caused by *Mycobacterium tuberculosis* [8], *Borrelia burgdorferi* [9], *Pseudomonas aeruginosa* [3], and uropathogenic *Escherichia coli* [10]. Persister cells are growth-arrested, but can restart growth when the external stress is removed, causing relapse of infection [5,10,11]. In addition, treatment of persistent infections results in overuse of antibiotics, contributing to the development of antibiotic resistance through mutations [12]. Therefore, a strategy to eradicate persister cells is urgently needed.

Molecular mechanisms leading to persister formation have been associated with the toxin-antitoxin (TA) modules, which are small operons that encode a toxin protein and its corresponding antitoxin [13]. An imbalance between the TA modules results in persister formation with arrested growth. Six types of TA systems have been reported to date [14]. The best known example of TA systems is the *E*. *coli* HipAB toxin/antitoxin pair, which encodes the toxin HipA and antitoxin HipB [15]. It was the first module found to play an important role in persister formation, and thus, the name *high incidence of persistence*. Since then, many TA systems have been discovered in bacteria including major pathogens such as *M. tuberculosis* [16], *P*. *aeruginosa* [17], and *Staphylococcus aureus* [18].

Persister cells are metabolically inactive, and thus lack growth-associated targets of most antibiotics [5–7]. One possible strategy to overcome the challenge of persistence is to identify agents that can kill the persister population directly. Mitomycin C [19] and cisplatin [20] have been shown to crosslink the DNA and kill persister cells. Specifically, mitomycin C can enter cell passively and crosslink guanine bases on different DNA strands [20,21], while cisplatin crosslinks the purines. In addition, cisplatin contains a platinum ion, which may contribute to the production of ROS [20]. Mitomycin C showed promising activities for topical use in an in vitro wound infection model [19]. Meanwhile, there are reports of toxicity of cisplatin and mitomycin C at high concentrations when administered intravenously for cancer treatment [22–24].

With direct killing of persister cells being difficult, another strategy that has been explored is to address the challenge of dormancy associated reduction of antibiotic penetration. Gram-negative bacteria are particularly challenging due to the presence of an outer membrane (OM) composed of anionic lipid polysaccharides [25]. In general, hydrophilic antibiotics can gain access to the cell interior through porins in the OM, while hydrophobic molecules enter through the lipid bilayer [25]. Dormancy is accompanied by significant reduction in membrane potential [26–28], which blocks the penetration of antimicrobials that rely on active uptake. Even for antibiotics that enter cells by energy-independent diffusion through porins, such as β-lactams, the decrease in membrane potential reduces the ion motive force for positively charged molecules, making it less favorable for drug influx [29].

A few strategies have been reported to promote penetration of antibiotics, primarily aminoglycosides, into persister cells. These strategies include increasing aminoglycoside uptake through hypoionic shock [30], generating proton motive force (PMF) with metabolites [31], and conjugating tobramycin with a membrane targeting peptide [32]. For example, Allison et al.[31] demonstrated that it is possible to kill persister cells with internalized gentamycin during wake-up with resumed central metabolism (with carbon source such as glucose, pyruvate, mannitol, or fructose), but not full growth activities. However, these strategies require potentiation with sugar or hypoionic shock, which can be difficult to apply *in vivo*.

Motivated by these challenges, we aim to identify effective control agents that do not require pretreatment. We started this study by testing tetracycline and minocycline, both are from the tetracycline family. We chose this family of antibiotics because they all target the 30S ribosome subunit, but have different binding affinities and membrane penetration capabilities. Thus, the effects of different factors can be compared. Both tetracycline and minocycline are substrates of *E*. *coli* major facilitator superfamily (MFS) and resistance-nodulation-cell division (RND) efflux pumps [33] and thus are ineffective against the normal cells of *E*. *coli*. However, since both types of efflux pumps require proton motive force (PMF) to function, we hypothesize that efflux would be inactive in persisters, providing favorable conditions for antibiotic accumulation and persister killing during wake-up. Here we present results that support this hypothesis and demonstrate that minocycline is effective in killing *E*. *coli* persister cells. This led to a set of principles for identifying persister control agents based on this mechanism, which should (1) be positively charged under physiological condition to interact with the negatively charged lipopolysaccharides on bacterial outer membrane, (2) be amphiphilic to have membrane activity for penetration, (3) be capable of penetration by energy-independent uptake, (4) have strong binding affinity with the target. We validated these principles by testing eravacycline, which has stronger binding to its target than minocycline, and found eravacycline to be more potent in killing *E*. *coli* persister cells than minocycline (3 logs vs. 0.5 log of killing at 100 μg/mL).

## Results

### Minocycline is effective in killing persister cells but not normal cells of *E*. *coli*

It is generally believed that conventional antibiotics that can kill normal cells are ineffective against persister cells [6]. To test our hypothesis and identify persister control agents, we took a different approach to test antibiotics that are ineffective against normal cells and are substrates of drug efflux pumps. We first tested tetracycline and minocycline, both from the tetracycline family of antibiotics. Both antibiotics target protein translation by binding to the ribosome complex [34–37] and are substrates of the RND and MFS efflux pumps [33,38]. These efflux pumps require proton motive force to function and are involved in pumping out multiple agents such as antibiotics and toxins [35]. Thus, we speculate that these compounds will accumulate more in persister cells than normal cells.

**Fig 1:**
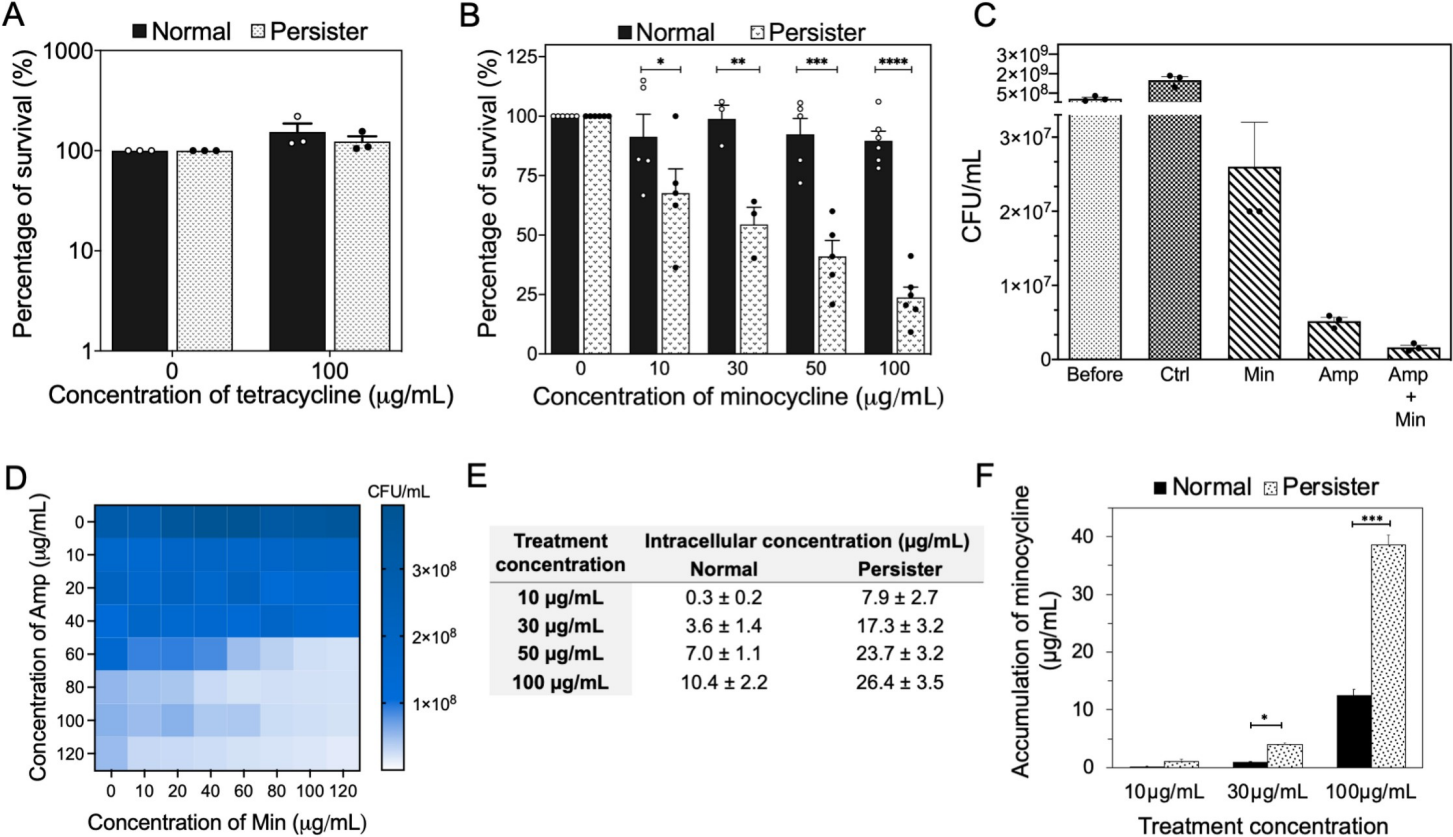
Minocycline is more effective against *E*. *coli* HM22 persister cells than normal cells. (A) Viability of *E*. *coli* HM22 persister and normal cells after tetracycline treatment in PBS. (B) Effects of minocycline (in PBS) on the viability of normal (black bars) and persister (white bars) cells of *E*. *coli* HM22. The untreated samples from each population were normalized as 100%. Means ± SE are shown (n = 5). (C) Different antibiotic treatments of *E*. *coli* HM22 persister cells including 100 μg/mL of ampicillin, 100 μg/mL of minocycline, and the combination of both treated in LB medium. (D) The effects were corroborated by a checkerboard assay. The exponential phase cells were treated with ampicillin for 3 h in LB, followed by treatment with minocycline for 1 h in PBS. (n = 2) (E) Intracellular concentration of minocycline based on the reporter bioassay. Minocycline concentration was calculated using a standard curve of reporter strain for each population (S1 Fig). Means ± SE are shown (n = 4). (F) Intracellular concentration of minocycline from treated and untreated samples in both normal (black bars) and persister (patterned bars) populations using LC-MS. Means ± SE are shown (n = 3). * *p*-value≤ 0.05, ** *p*-value ≤ 0.01, *** *p*-value ≤ 0.001, ****p-value ≤ 0.0001.

*E*. *coli* HM22 was used as the model strain in this test because it contains the *hipA7* allele that leads to high-level persistence [15,39–44]. First, we treated *E*. *coli* HM22 cells in exponential phase (~99% as normal cells [15,39]) and persister cells isolated with ampicillin. Both exponential phase cells and persister cells were tolerant to tetracycline (Fig 1A). However, they responded differently to minocycline treatment (in PBS). As expected, even at a high concentration of 100 μg/mL, there was no significant killing (Fig 1A) of normal cells (cells from exponential cultures) by minocycline. In contrast, exposure to minocycline killed 32.3 ± 9.1% (*p* = 0.030), 47.8 ± 5.3% (*p* = 0.047), 59.0 ± 6.0% (*p*<0.001), and 70.8 ± 5.9% (*p*<0.001) of isolated persister cells when treated at 10, 30, 50, and 100 μg/mL, respectively (Fig 1B). This demonstrated persister killing by minocycline in a dose-dependent manner. Because the persister cells were isolated using ampicillin, we further evaluated if ampicillin played a role in the increased killing of persister cells by minocycline. To test this, we compared the treatment with 100 μg/mL ampicillin alone and concurrent treatment with both 100 μg/mL ampicillin and 100 μg/mL minocycline in LB. The results showed that adding minocycline caused an additional 68% of killing (*p* = 0.0038, unpaired t-test) (Fig 1C). This finding confirms that minocycline does have significant killing effects on persister cells. To further corroborate the results, we conducted a checkerboard assay to treat with various concentrations of ampicillin for 3 h in LB medium first, followed by different concentrations of minocycline for 1 h in PBS after ampicillin removal. When treated with low concentrations of ampicillin (e.g. less than 60 μg/mL), minocycline was not effective. This is expected because the concentration of ampicillin was not enough to kill all normal cells. After treatment with higher concentrations of ampicillin, dose-dependent killing by minocycline was observed. These results corroborate the effects of minocycline in persister killing (Fig 1D).

### Persister cells accumulate more minocycline intracellularly than normal cells

It is interesting that tetracycline and minocycline have different activities against *E*. *coli* persister cells although they are from the same antibiotic class. To further understand the stronger killing efficacy of persister cells than normal cells by minocycline, we quantified the intracellular concentration of minocycline in these two populations. Two complementary approaches were used for this test, including a new reporter strain-based bioassay we developed recently [45] and conventional LC-MS analysis (S1 Fig). The reporter assay is based on *Bacillus subtilis* 168, which is susceptible to minocycline and allows the quantification of minocycline in *E*. *coli* cell lysate by fitting in the standard curve (S1 Fig). Using this assay, we quantified the intracellular concentration of minocycline to be 10.4 ± 2.2 and 26.4 ± 3.5 μg/mL in normal and persister cells, respectively, after treatment with 100 μg/mL minocycline for 1 h (Fig 1E). The findings indicate that *E*. *coli* persister cells accumulated ~2.6 times the intracellular concentration of minocycline compared to normal cells. This finding was corroborated by LC-MS analysis, which revealed persister cells to harbor 3.0 ± 0.4 times the intracellular concentration of minocycline relative to normal cells, after the same treatment (*p*<0.0001) (Fig 1F). In comparison, the opposite was found for tetracycline. Specially, the intracellular concentration of tetracycline was found to be 5.5 ± 0.1 and 1.6 ± 0.8 μg/mL in normal and persister cells, respectively, after the same treatment at 100 μg/mL.

The difference in antibiotic accumulation between tetracycline and minocycline is not unexpected. Tetracycline uptake can occur by diffusion but mostly through energy-dependent mechanisms [46] while minocycline enters bacterial cells mainly by passive diffusion [35,47,48]. In addition, tetracycline has a lower binding affinity to the target compared to minocycline. The dissociation constant of minocycline and 30S ribosome subunit is 3.5x10^-7^ M [49]. In comparison, the dissociation constant between tetracycline and its target is 1.3x10^-5^ M [49], approximately two orders of magnitude higher than minocycline. Collectively, our results indicate that persister killing by minocycline but not tetracycline was due to higher accumulation and stronger target binding of minocycline.

### Persister cells have reduced efflux activities

**Fig 2:**
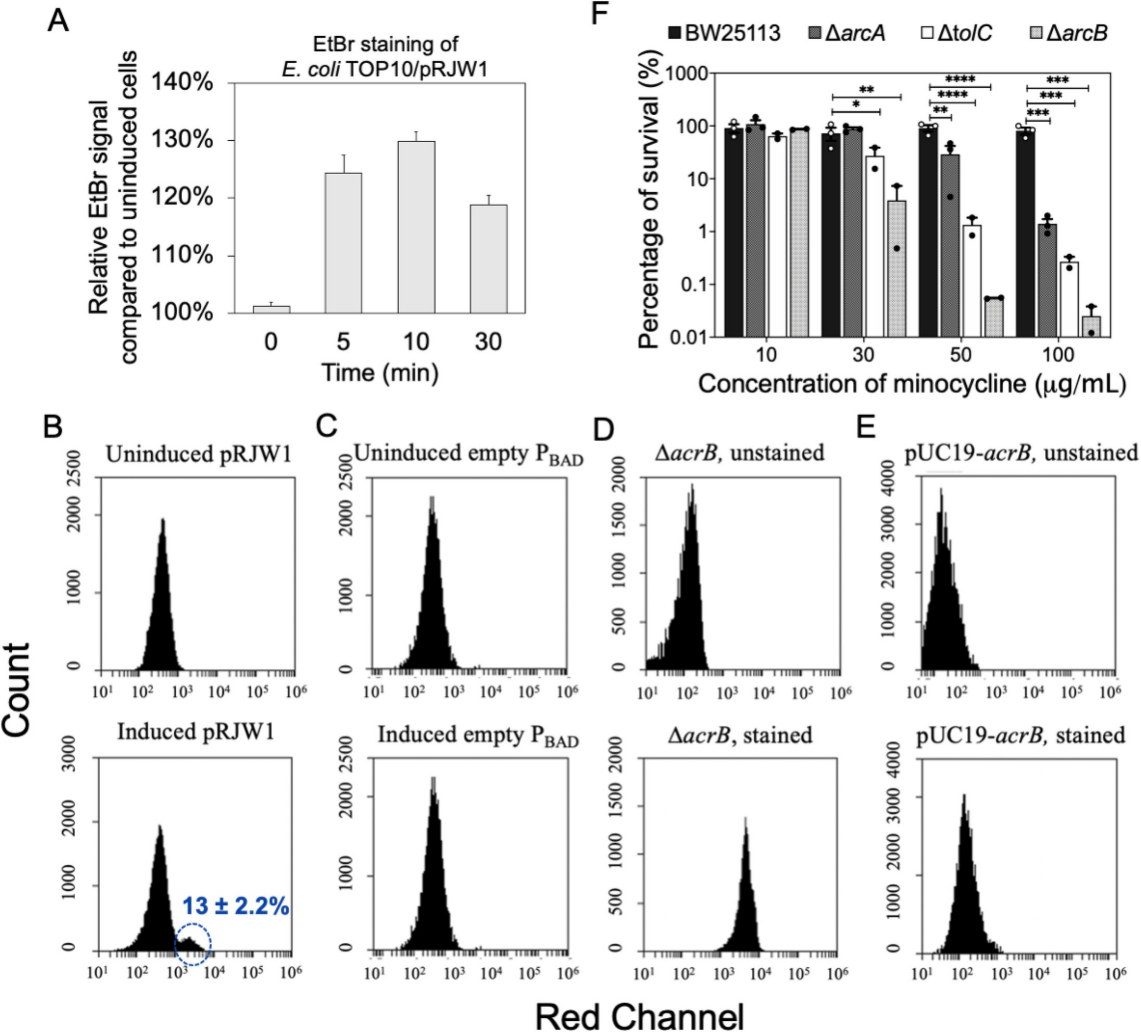
Persister formation led to reduced efflux pump activities. (A) Induction of persister formation led to increased EtBr accumulation. Measurements were performed with a fluorescence microplate reader with excitation at 360 nm. (B-E) Flow cytometry analysis of EtBr staining. (B) EtBr stained uninduced *E*. *coli* Top10/pRJW1 (top) and EtBr stained arabinose induced *E*. *coli* Top10/pRJW1 (bottom). Induced *E*. *coli* Top10/pRJW1 had 13 ± 2.2% of the population shifted to stronger fluorescence, indicating more EtBr accumulation. (C) EtBr stained uninduced *E*. *coli* Top10 pBAD (top) and EtBr-stained arabinose induced *E*. *coli* Top10/pBAD (empty vector) (bottom). (D) *E*. *coli* Δ*acrB* without (top) and with (bottom) EtBr staining. (E) *E*. *coli* pUC19-*acrB* without (top) and with (bottom) EtBr staining. (F) Inactivation of efflux pumps sensitized normal cells to minocycline. Means ± SE are shown (n = 3). * *p*-value≤ 0.05, ** *p*-value ≤ 0.01, *** *p*-value ≤ 0.001, ****p-value ≤ 0.0001.

Because minocycline is a substrate of the RND and MFS efflux pumps [35,47,50–52], we hypothesized that increased accumulation of antibiotics such as minocycline in persister cells is linked to reduced efflux activities. To test this hypothesis, we compared normal and persister cells of *E*. *coli* using ethidium bromide (EtBr) staining since the concentration of EtBr in bacterial cells is determined by efflux activities driven by PMF [53]. To avoid the interference of signals from dead cells and cell debris, we used the *P*_*BAD*_ inducible system to generate persisters in this experiment rather than persister isolation by killing normal cells using ampicillin. To do so, *E*. *coli* Top10/pRJW1 was constructed to allow *hipA* overexpression under the control of the arabinose-inducible *P*_*BAD*_ promotor. The EtBr signal increased in cells exposed to arabinose (to induce *hipA* expression and thus persister formation) relative to the uninduced samples. Specifically, the EtBr signal was 24.3 ± 3.2%, 29.9 ± 1.6%, and 18.9 ± 1.5% higher in induced relative to uninduced samples after 5, 10, and 30 min of incubation, respectively (Fig 2A. *p*<0.001 for all conditions). The difference in EtBr accumulation decreased after 30 min of incubation is possibly due to the toxicity of EtBr. These results were confirmed using flow cytometry (Fig 2B–2E). When the *hipA* gene was overexpressed, approximately 13 ± 2.2% of the population shifted further to stronger red fluorescence compared to the uninduced control (Fig 2B–2C). To verify if this shift was due to decrease in efflux pump activities, we repeated the EtBr staining using an efflux mutant *E*. *coli* Δ*acrB*. As shown in S1 Fig, stained normal cells of *E*. *coli ΔacrB* exhibited strong fluorescence (Fig 2D) similar to the brightest subpopulation of induced pRJW1 cells (Fig 2B), which was not observed in the complemented strain (Fig 2E). This finding strongly suggests that persister cells have reduced efflux activities.

### Inactivation of efflux pumps sensitized normal cells to minocycline

Minocycline is not effective in killing *E*. *coli* normal cells due to drug efflux by RND and MFS pumps [35,47,50–52], both require PMF to function. Since persister cells that are sensitive to minocycline demonstrated reduced efflux activity, we next asked if inactivating or reducing efflux activity in normal cells would render normal cells as sensitive to killing by minocycline as persister cells. To test this, *E*. *coli* JW4364 (*ΔacrA* mutant), JW5536 (*ΔacrB* mutant), and JW5503 (*ΔtolC* mutant) were compared with their wild-type strain *E*. *coli* BW25113 for minocycline susceptibility. As expected, we observed increased killing of all three efflux mutants compared to the wild-type strain. For example, 100 μg/mL minocycline killed normal cells of *ΔacrA*, *ΔacrB*, and *ΔtolC* by 98.6 ± 0.3% (*p* = 0.0002), 99.9 ± 0.01% (*p* = 0.0002) and 99.7 ± 0.03% (*p* = 0.0011), respectively; while no significant killing of normal cells of the wild-type strain was observed (Fig 2F). This finding further demonstrates a correlation between the lack of efflux and increase in persister killing.

### *E*. *coli* persister cells have lower membrane potential than normal cells

**Fig 3:**
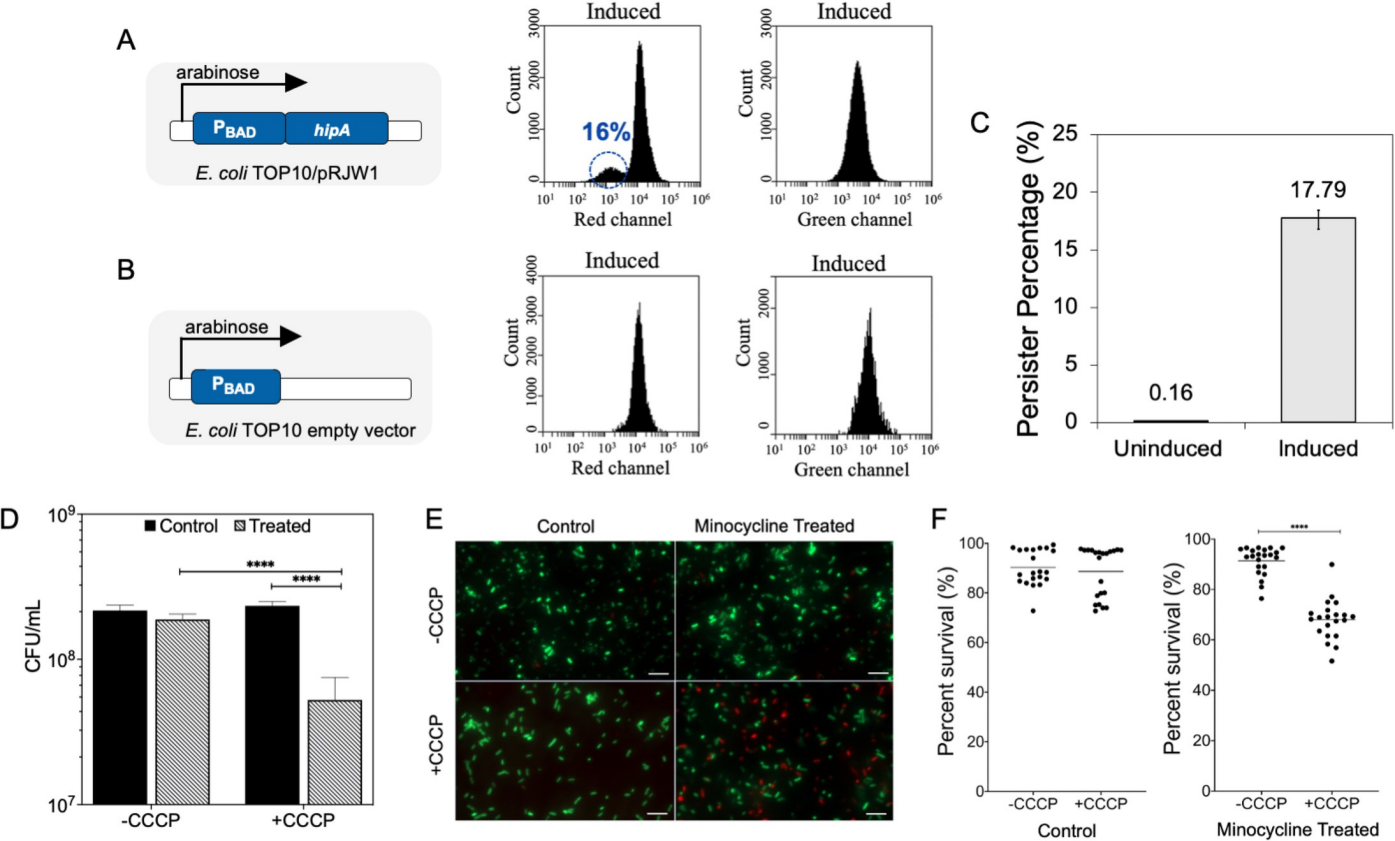
Persister formation led to lower membrane potential. (A, B) Schematic of *hipA* mediated persister formation by the P_BAD_ protmoter. Flow cytometry analysis of JC-1 stained samples was used to compare the membrane potential of induced (with arabinose and tetracycline) *E*. *coli* Top10/pRJW1 (A) vs. induced *E*. *coli* Top10/pBAD (empty vector) (B) cells. A shift to low red fluorescence was observed for 16 ± 2.5% of induced cells of *E*. *coli* Top10/pRJW1, while no change was observed in green fluorescence. (C) Persister count increased when induced with both arabinose and tetracycline. (D) *E*. *coli* HM22 normal population was pretreated with CCCP (100 μM) to reduce the membrane potential. The cells were then treated with 100 μg/mL of minocycline. Means ± SE are shown (n = 3). (E) Representative fluorescent images of control and minocycline treated *E*. *coli* HM22 normal cells with or without CCCP pretreatment. The cells were labeled with SYTO9 and propodium iodide (PI) (scale bar, 10 μM). (F) Cell viability based on mean fluorescence intensity quantified using Image J. Percentages are based on the ratios of green fluorescence vs. total fluorescence. * *p*-value≤ 0.05, ** *p*-value ≤ 0.01, *** *p*-value ≤ 0.001, ****p-value ≤ 0.0001.

Previous studies have reported the association between persister formation and the reduction of membrane potential [26,27]. For example, pretreatment with salicylate collapses the membrane potential through the production of ROS [26]; thereby inducing persistence. In addition, increase in Obg levels induces the production of HokB, a small membrane peptide, that induces persistence through pore formation leading to ATP leakage and membrane depolarization [27,54]. These can explain the reduced efflux activities in persister cells observed in our study. To confirm if our persister cells also have lower membrane potential than normal cells, we compared *E*. *coli* Top10/pRJW1 normal and persister cells using JC1, a potentiometric dye that has the ratio of red/green fluorescence positively correlated with membrane potential. Upon induction of persister formation by overexpressing *hipA*, approximately 16 ± 2.5% of the total counts (Fig 3A) exhibited a reduction in the red fluorescence (no change in green fluorescence), while the rest of the population had a strong red fluorescence as observed in the uninduced control (Fig 3B). It is of interest to note that induction of persister formation by *hipA* overexpression coincided with 17.8 ± 0.6% (Fig 3C) of the induced population as persister cells, as confirmed by CFU counts. This suggests that the shift toward lower red fluorescence, and thus, reduced membrane potential in 16% of the cell population likely occurred in persister cells.

### Membrane depotentiation leads to increased killing of *E*. *coli* cells by minocycline

The above results indicated that reduced efflux activity in persister cells can lead to increase in accumulation of certain antibiotics like minocycline. If increased accumulation of antibiotics is indeed the cause of persister killing, we anticipated that membrane depotentiation will likewise sensitize normal cells to minocycline. Because the membrane potential is governed by the PMF and transmembrane pH gradient across the bacterial cell membrane [55], a reduction in membrane potential indicates reduced PMF which impairs the function of efflux pumps. This leads to increased accumulation of antibiotics that penetrate bacterial membranes without active transport, such as minocycline. We therefore made use of carbonyl cyanide m-chlorophenylhydrazone (CCCP) [56] to depotentiate the membrane of *E*. *coli* normal cells and thus, mimic the change in membrane potential of persister cells. CCCP dissipates the PMF by allowing protons to leak across the membrane [57] and thus inactivates efflux pumps that require PMF to function. Previous studies have shown that CCCP treatment enhances persister formation in *E*. *coli* and *P*. *aeruginosa* [58–60]. We first pretreated the normal cells with 100 μM of CCCP for 10 min, followed by treatment with 100 μg/mL of minocycline for 1 h. This led to 95.9 ± 2.5% (*p* = 0.0146) killing of *E*. *coli* normal cells. In contrast, no significant killing by minocycline was observed in the absence of CCCP pretreatment (*p* = 0.9084) (Fig 3D). The CFU results were corroborated by LIVE/DEAD staining that showed significant increase in red fluorescence (propidium iodide stains cells with compromised membranes) among cells treated with minocycline after CCCP pretreatment, while the controls (minocycline alone without CCCP) showed little to no red fluorescence (Fig 3D–3F). It is worth noticing that the image still shows a large number of green cells. This is because the images were taken while the cells were in PBS, which does not have carbon sources to support growth. The majority of killing occurred after the cells were plated on LB agar plates (as shown in CFU results), which supports cell growth and thus the full strength of killing.

### Killing of persister cells occurs during wake-up

**Fig 4:**
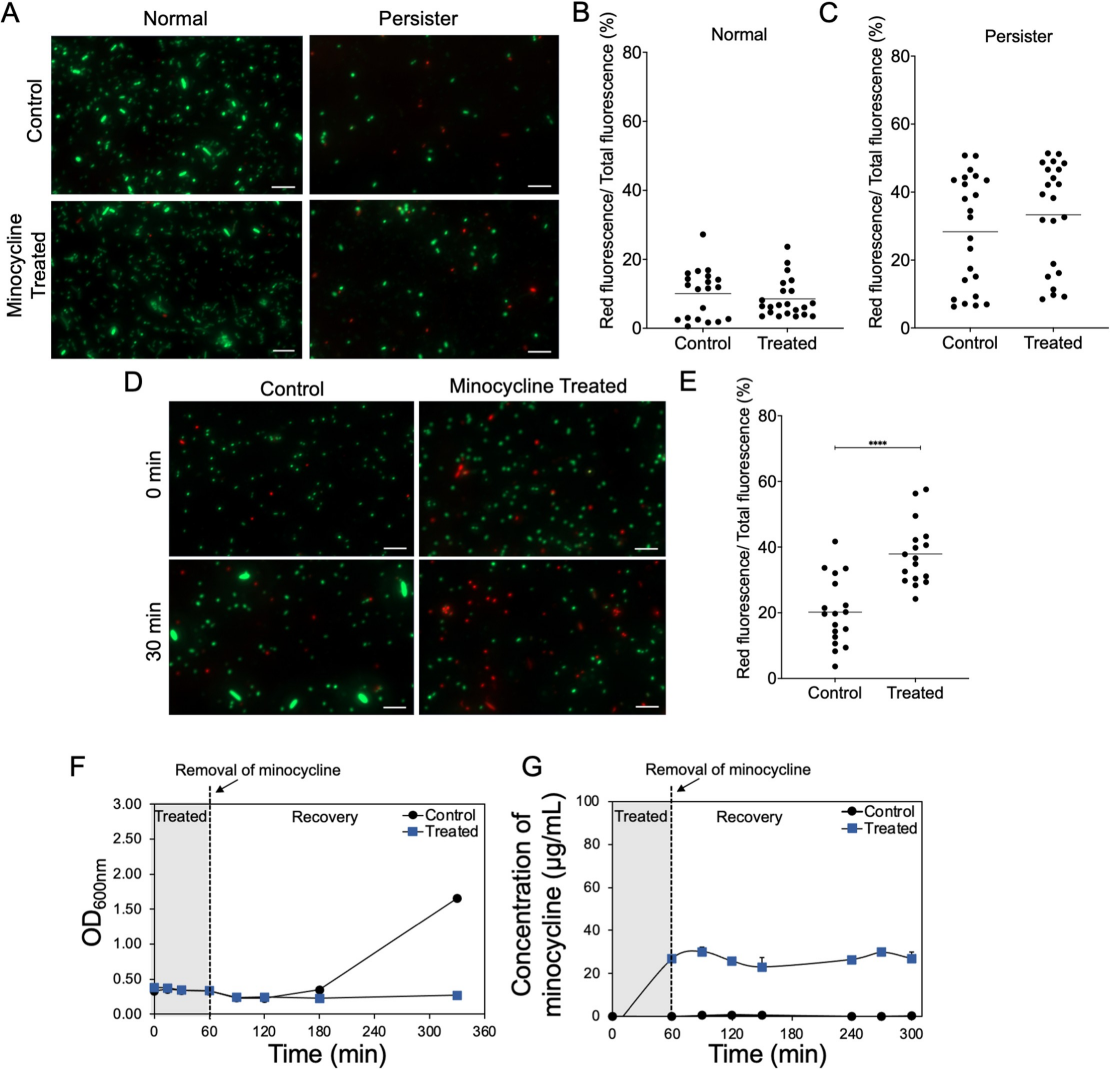
Killing occurred upon wake-up of persister cells. (A) Representative fluorescence images of control and minocycline treated *E*. *coli* HM22 normal and persister cells after LIVE/DEAD staining (scale bar = 10 μm). Fluorescence signals were used to compare the viability of normal (B) and persister (C) cells. Mean fluorescence intensity of SYTO9 and PI was quantified using ImageJ. (D) Representative fluorescence images of persister cells upon wake-up after minocycline treatment. The images show persister and normal cells at 0 and 30 min after spiking with LB medium. (E) Fluorescence signals of LB spiked *E*. *coli* HM22 persister cells. Three biological replicates were tested with 16 images randomly analyzed from each sample. (F) OD_600_ of *E*. *coli* HM22 persister cells during wake-up. Cells with and without minocycline treatment were compared. (G) Intracellular concentration of minocycline after minocycline was removed from the solution and replaced with LB medium. * *p*-value≤ 0.05, ** *p*-value ≤ 0.01, *** *p*-value ≤ 0.001, ****p-value ≤ 0.0001.

Although persister cells accumulate more minocycline, these cells are dormant and thus lack the growth-associated activities needed to generate corrupted products for killing to occur. We therefore speculated that the killing effects took place during persister wake-up when the external antibiotic was withdrawn (after the treated cells were plated on antibiotic-free agar plates in this test). Minocycline has a dissociation constant of 3.5 x 10^−7^ M to the 30S subunit of ribosome [49]; thus, we speculate that when the extracellular concentration of minocycline decreases there is still sufficient intracellular antibiotic concentration to kill these cells upon wake-up. To understand if this occurs, we followed the dynamic change of the viability of *E*. *coli* HM22 persister cells after extracellular minocycline was removed, and nutrients were added to “wake up” persister cells. The persister population showed stronger red fluorescence after LIVE/DEAD staining than normal cells in general, presumably due to reduced membrane potential and higher permeability to propidium iodide (Fig 4A). But no significant difference (*p* = 0.4423) in red/total fluorescence ratio was observed before and immediately after minocycline treatment (100 μg/mL in PBS) (Fig 4A–4C). This result indicates that the killing of persister cells did not occur during the 1 h minocycline treatment.

To test if killing occurred during persister wake-up, the untreated and treated samples were then replenished with 500 μL of LB after washing the cells with PBS to remove extracellular minocycline. After 30 min of incubation with added LB, the red/total fluorescence in untreated persisters decreased from 28.3 ± 4.8% to 20.9 ± 3.3%, indicating the cells were waking up. In contrast, the minocycline treated cells exhibited an increase in red fluorescence from 20.2 ± 3.3% to 37.9 ± 3.0%. The different trends between the two groups (Fig 4D–4E) indicate that killing occurred during wake-up. Consistently, the untreated persister population gradually regrew with an increased OD_600_ at 2 h after adding LB medium, while the OD_600_ of the treated population remained the same (Fig 4F). This is consistent with the CFU data in Fig 1B and the fluorescence results in Fig 4D. Using the same reporter assay, we found that the minocycline concentration remained at 26.8 ± 3.2 μg/mL in persister cells even 4 h after washing the cells and adding LB medium (Fig 4G), essentially unchanged from 26.9 ± 0.7 μg/mL right after the 1 h treatment (Figs 4G and 1D). To corroborate these results, we monitored the efflux activity using a *tolC* reporter from the *E*. *coli* promoter-GFP fusion library [61], which has each individual gene promoter fused with a promoterless *gfp* gene. We chose this reporter because minocycline is a substrate of the AcrAB-TolC efflux pump [33]. We isolated persister cells and monitored the GFP expression during wake-up. The results indicated that while persister cells resumed growth within the 2 h after wash and transfer to fresh LB medium, the expression of *tolC* took 4 h to become detectable (S3 Fig). This provides a window for the killing to occur if the cells are pre-treated with minocycline, as we observed in this study (Fig 1). This is also consistent with the finding that minocycline concentration inside persister cells remained rather constant during this period, further supporting the hypothesis. Collectively, these findings provide a better understanding of the kinetics of efflux pump activities during persister wake-up. These results show that removing the extracellular antibiotic and adding nutrients to wake up persister cells can cause killing of this dormant population by internalized antibiotics if there is strong binding to the target.

### New criteria for selecting persister drugs

**Fig 5:**
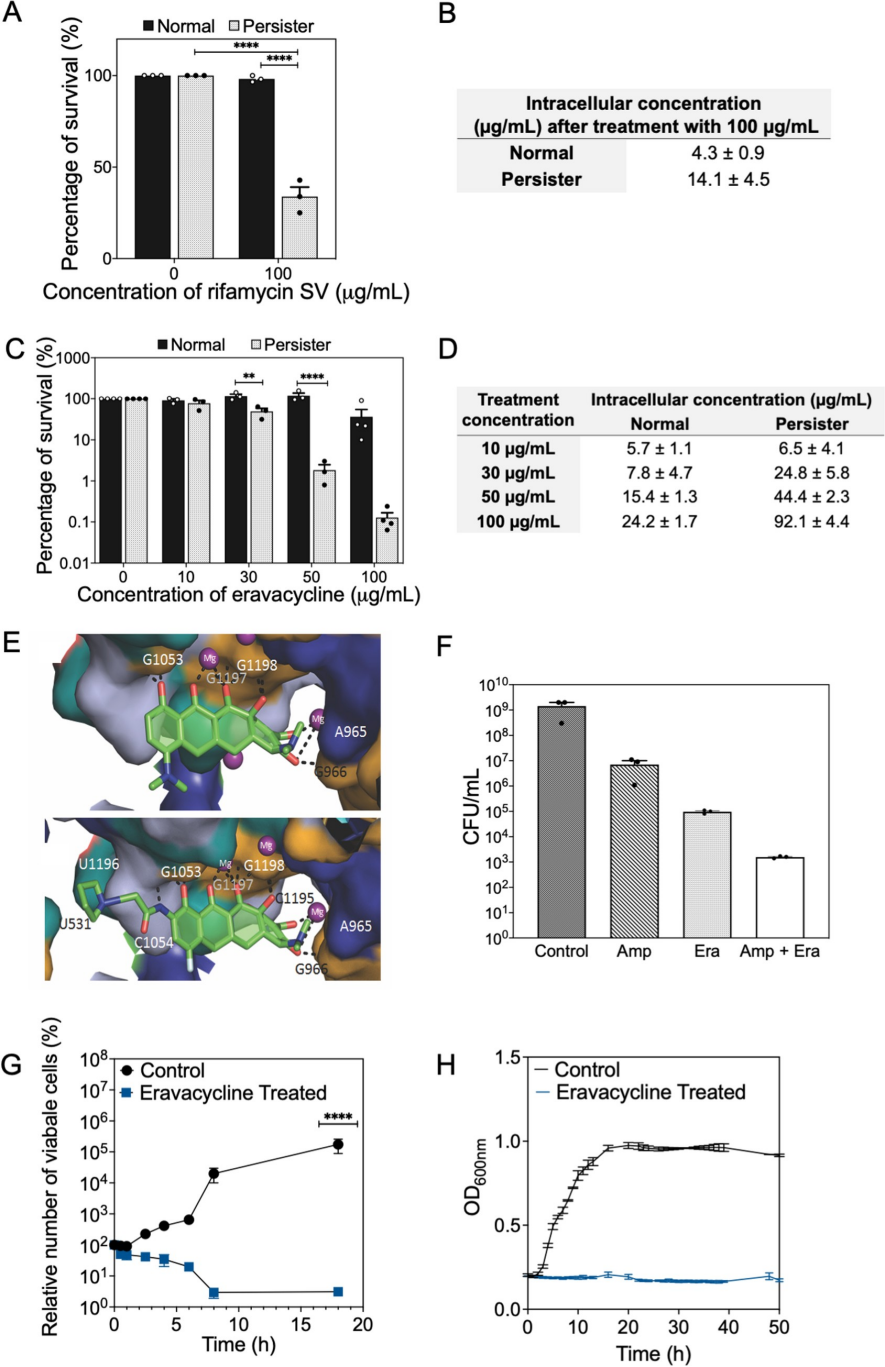
Viability of *E*. *coli* HM22 after treatment with rifamycin SV and eravacycline. (A) Effects of 100 μg/mL rifamycin SV on the viability of normal (black bars) and persister (patterned bars) cells of *E*. *coli* HM22. Means ± SE are shown (n = 3). (B) Intracellular concentration of rifamycin SV based on the reporter bioassay. Rifamycin SV concentration was calculated using the standard curve of reporter strain for each population (S2B Fig). Means ± SE are shown (n = 3). (C) Effects of eravacycline on the viability of normal (black bars) and persister (patterned bars) cells of *E*. *coli* HM22. Means ± SE are shown (n = 3). (D) Intracellular concentration of eravacycline based on the reporter bioassay. Eravacycline concentration was calculated using a standard curve of reporter strain for each population (S2C Fig). Means ± SE are shown (n = 3). (E) Binding pocket of minocycline (top) and eravacycline (bottom) in the 30S ribosomal unit. Minocycline interacts with G966, C1195, U1196, G1197, and G1198 via hydrogen bonding and ionic interactions (black dashed lines) mediated by a pair of Mg ions. Eravacycline occupies the same binding pocket as minocycline but also binds to additional residues—C1054, C1195, and U1196. (c) Color scheme: uracil (teal), cytosine (light blue), guanine (gold), adenine (purple), C (green stick); O(red, stick); N (blue); F (cyan, stick); Mg ion (magenta, sphere). Solvent is omitted for clarity. (F) Different antibiotic treatments of *E*. *coli* HM22 persister cells including 100 μg/mL of ampicillin, 100 μg/mL of eravacycline, and the combination of both. Means ± SE are shown (n = 3). (G-H) Relative number of viable *E*. *coli* HM22 persister cells after eravacycline treatment (initial number normalized as 100%). The changes in OD_600_ (G) and CFU (H) were followed over time. Means ± SE are shown (n = 3 for OD_600_ and n = 4 for CFU). * *p*-value≤ 0.05, ** *p*-value ≤ 0.01, *** *p*-value ≤ 0.001, ****p-value ≤ 0.0001.

Based on these results, we developed a set of criteria for selecting persister control agents. Specifically, a good persister drug should: (1) be positively charged under physiological condition to interact with the negatively charged lipopolysaccharides on bacterial outer membrane, (2) be amphiphilic to have membrane activity for penetration, (3) be capable of penetration by energy-independent uptake, (4) have strong binding affinity with the target. The first three criteria will ensure effective penetration and accumulation; while (4) is important for killing to occur when persisters wake up with the withdrawal of extracellular antibiotics (killing occurs before the antibiotic is extruded or diffuses out). To validate this strategy, we tested rifamycin SV, a hydrophobic antibiotic that penetrates Gram-negative cells by diffusion through lipid bilayers and targets the RNA polymerase [25,62]. In addition, rifamycin SV is a substrate of the RND efflux pump encoded by AcrAB-TolC [33]. Our data indicate that 100 μg/mL rifamycin SV did not kill normal cells but killed 75.0 ± 5.12% of persister cells (*p*<0.0001; Fig 5A). In addition, persister cells accumulated 3.2 times of this antibiotic compared to normal cells (14.1 ± 4.5 vs. 4.3 ± 0.9 μg/mL) when both populations were treated with 100 μg/mL rifamycin SV (Fig 5B).

Based on these principles, we furthermore tested eravacycline, a derivative of minocycline recently approved by FDA in 2018 [63]. Eravacycline also targets the 30S subunit of the ribosome; however, unlike minocycline, the pyrrolidinoacetamido group at C-9 position of eravacycline [64] forms an additional bond with the ribosome [65–68]. Eravacycline has also been reported to be more potent in inhibiting the ribosomes compared to tetracycline based on their IC50 (concentration of the antibiotic needed to inhibit 50% of the purified 70S ribosome), e.g., 0.2 ± 0.1 μM (eravacycline) vs. 3.0 ± 1.2 μM (tetracycline) [64]. To understand if additional binding to the 30S subunit can increase persister killing, we tested eravacycline at 0, 10, 30, 50, and 100 μg/mL by following the same experimental protocol as other antibiotics in this study. At concentrations between 0–50 μg/mL, there was no significant killing (Fig 5C) of normal *E*. *coli* HM22 cells; while it killed 63.5 ± 15.8% at 100 μg/mL (*p* = 0.0043). In a sharp contrast, it killed 50.9 ± 7.7% (*p* = 0.06), 98.2 ± 0.5% (*p*<0.001), and 99.9 ± 0.03% (*p*<0.001) of persister cells (Fig 5C) at 30, 50, and 100 μg/mL, respectively. Thus, 3 logs of killing of persister cells was achieved at 100 μg/mL. Similar to minocycline and rifamycin SV, persister cells also accumulated more eravacycline than normal cells. For example, after treatment with 100 μg/mL eravacycline, persister cells accumulated 92.1 ± 4.4 μg/mL, 3.8 times of that in normal cells (24.2 ± 1.7 μg/mL; Fig 5D).

To obtain more insights into the difference between eravacycline and minocycline in target binding and thus the activities of persister killing, we conducted molecular dynamics simulation to compare the docking of these two antibiotics. Eravacycline and minocycline have the same binding pocket in the 30S ribosomal subunit (Fig 5E). The binding pocket is lined by G966 C1195, G1053, G1197, and G1198 mRNA residues. In minocycline, the hydroxyl and carbonyl groups bind to the pocket via hydrogen bonds or ionic interactions mediated by a pair of magnesium ions. In eravacycline, the additional pyrrolidinoacetamido group at C9 interacts with C1054 and U531 to provide extra stability compared to minocycline [66]. Additionally, in our molecular dynamics simulation, we observed ionic interaction of the fluorine, located at C-7 position of eravacycline, with the solvated Mg ions in solution. The 2 μs simulation was not long enough to observe conformational changes in the eravacycline or the rRNA nucleotides, but this simulation validated the stronger binding of eravacycline compared to minocycline.

We further evaluated if ampicillin (used to isolate persister cells) played a role in the increased killing of persister cells by eravacycline. Similar to the test of minocycline above, concurrent treatment with both 100 μg/mL ampicillin and 100 μg/mL eravacycline caused an additional 99.9% of killing compared to the treatment with 100 μg/mL ampicillin alone (Fig 5F). These results were corroborated by comparing the IC50 (concentration required to kill 50% of the population) of individual treatments vs. co-treatment. The IC50 values were found to be 1.0 μg/mL (ampicillin alone), 4.8 μg/mL (eravacycline alone) and 0.1 μg/mL (concurrent treatment). This leads to a combination index [69] of 0.12, demonstrating strong synergy in bacterial killing. In addition, since both minocycline and rifamycin SV are substrates of the *E*. *coli* AcrAB-TolC pumps, we tested eravacycline on AcrAB-TolC mutants and found more killing of normal cells of these mutants compared to the wild-type (S4 Fig).

Consistent with the strong target binding of eravacycline, we found that the treated persister cells were unable to resume growth after removal of extracellular eravacycline and addition of nutrient (CFU continued to decrease by 97.1 ± 0.9% over 8 hours) while untreated persister cells regrew (Fig 3G–3H). Collectively, these results support our proposed criteria and demonstrate that potent killing of *E*. *coli* persister cells can be achieved by antibiotics that can penetrate bacterial membranes without active uptake and have strong binding to their target.

Beside the lab strains discussed above, we further tested this strategy on uropathogenic *E*. *coli* (UPEC), the leading causative agent of urinary tract infections (UTIs) and catheter-associated UTIs [70]. We treated both exponential phase cells and persister cells of UPEC with increasing concentrations of eravacycline. The results showed significant killing against both populations, e.g., 27.8 ± 3.8% (p = 0.02), 47.8 ± 15.4% (p<0.001), 85.4 ± 4.2% (p<0.001), 97.8 ± 0.7% (p<0.001) of normal cells, and 62.1 ± 8.2% (p<0.001), 75.6 ± 2.7% (p<0.001), 94.03 ± 1.7% (p<0.001), 99.9 ± 0.1% (p<0.001) of persister cells at concentrations of 10, 30, 50 and 100 μg/mL, respectively (S5A Fig). Besides planktonic cells, we also tested the effects of eravacycline on 48-h UPEC biofilms cultured on polydimethylsiloxane (PDMS; a polymer commonly used for manufacturing urinary catheters). Our results show that eravacycline can reduce UPEC biofilms by 95.8 ± 1.2% and 99.3 ± 0.5% (*p* = 0.01 for both), when treated at 50 and 100 μg/mL, respectively (S5B Fig).

## Discussion

While it is commonly stated that persister cells are tolerant to conventional antibiotics, our study reveals that these dormant cells can be killed by selecting the right antibiotics with appropriate treatment conditions. Specifically, we demonstrate that antibiotics capable of penetrating bacterial cells by energy-independent diffusion and binding to their target strongly can kill persister cells during wake-up. Thus, the decrease in membrane potential of persister cells provides an “Achilles’ heel” for killing this dormant population. For example, the binding of minocycline with the ribosome is an energy-independent process regardless of the cell’s physiological state [47,49,51,71]. This is expected to occur in persister cells due to the ability of minocycline to diffuse through the membrane and avoid extrusion due to reduced efflux activities in persisters that ultimately led to killing of this population. We provide evidence that the killing did not occur instantly but wake-up is required for the activity to take place (Fig 4). With the extracellular antibiotics removed and nutrients provided, persister cells can revert to normal cells, a process that requires transcriptional and translational activities. Minocycline and eravacycline both target the ribosome while rifamycin SV targets the RNA polymerase, leading to killing of persister cells during wake-up. This represents a new strategy for persister control (Fig 4).

A few criteria need to be met for this strategy of persister control to work. In general, the control agent needs to penetrate persister cells via energy-independent diffusion (amphiphilic compounds are favorable). The target should be present in persister cells; and the control agent should have sufficient binding affinity with the target so it does not diffuse out or gets extruded before killing occurs. Minocycline, rifamycin SV and eravacycline all meet these criteria. It is worth noticing that normal cells of *E*. *coli* are resistant to minocycline and rifamycin SV due to substrate specific efflux activities energized by membrane potential gradient. In general, this is not favorable for bacterial control. However, these antibiotics provide a promising solution to the challenges of persister cells. Besides PMF-driven efflux pumps, there are also PMF-independent efflux pumps such as ABC transporters that require ATP to function. Because persister cells have reduced ATP level [72], we speculate that this strategy may also work with agents that are substrates of those systems. This is beyond the scope of this study and is part of our ongoing work.

**Fig 6:**
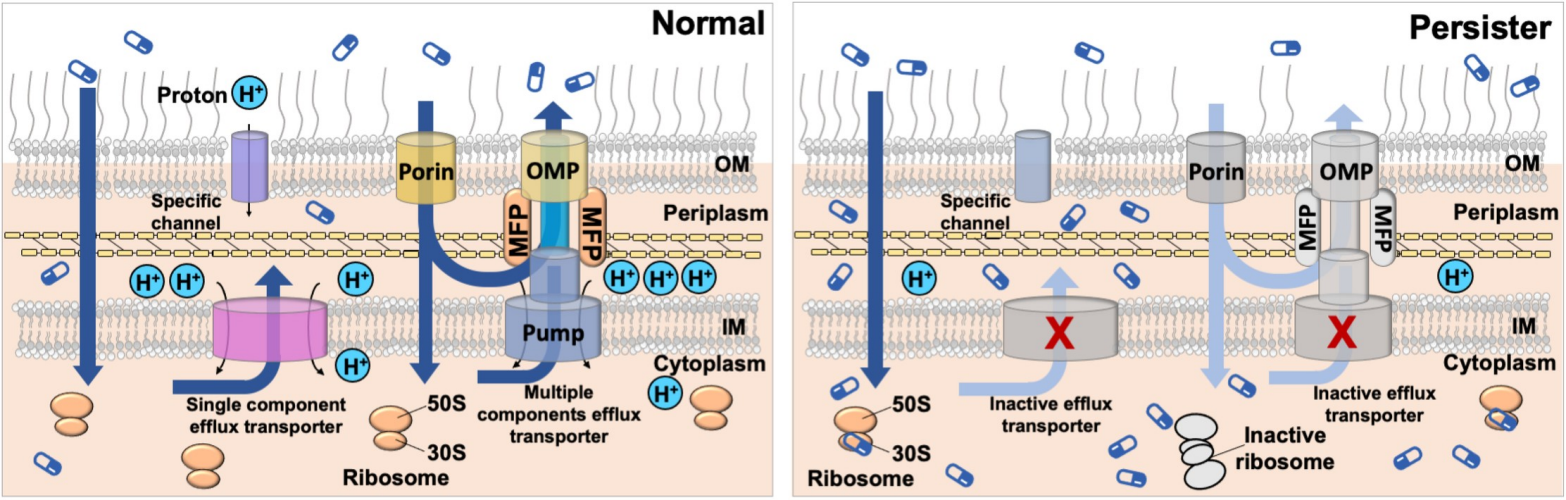
A conceptual model of persister control by leveraging reduced antibiotic efflux. Persisters have reduced membrane potential and thus are difficult to penetrate by hydrophilic antibiotics and those require active transport. In comparison, antibiotics that can penetrate through lipid without active uptake can still target persister cells. Additionally, reduced drug efflux provides a favorable condition for accumulation of antibiotics in persister cells. This leads to killing if the internalized antibiotic molecules remain bound to the target during wake-up. The inactivated pathways in persister cells are indicated with lighter colors and/or marked with “X”. Minocycline, rifamycin SV, and eravacycline fit the criteria and are found effective in this study for persister control. The drugs targeting the 30S ribosomal subunit demonstrated in this study are shown as an example. Figures are drawn for Gram-negative species as tested in this study.

The persister control strategy reported here is different from pulsed dosing of antibiotics that has recently been shown to improve the killing of biofilms and persister cells. It includes antibiotic-free periods between doses so that dormant cells can be killed after they resuscitate. However, pulsed dosing requires resuscitation but before overgrowth (to prevent the formation of new persisters), thus a narrow window between doses [73]. In comparison, the method reported here is a different strategy that targets persister cells specifically. It does not need repeated dosing and kills persister cells before they fully resuscitate due to increased accumulation and strong target binding in persister cells. It is also important to note that being the substrate of an efflux pump is just a factor that leads to different effects on normal (no killing due to efflux) and persisters (with killing due to reduced efflux). But this is not a prerequisite for persister killing. Drugs that satisfy the proposed set of criteria but are not substrates of efflux are expected to kill both normal and persister cells. As demonstrated in this study, eravacycline has killing effects on both populations although it is also more effective on persister cells as observed for minocycline and rifamycin SV. This antibiotic was designed to overcome resistance of common tetracycline-specific efflux [74]. A schematic summarizing this new strategy is shown in Fig 6.

The results from this study also emphasize the needs for new antibiotic discovery platforms. The vast majority of currently available antibiotics were discovered between 1940s-1960s using the Waksman platform [75]. In this approach, a possible source of antimicrobials (e.g. an soil sample containing *Actinomycetes*) is tested for its inhibiting zone on an overlay plate against a target bacterial species. This approach selects lead compounds based on growth inhibition and thus the hits commonly fail to achieve persister control. Based on the findings from this study, we believe future screenings based on membrane penetration may generate new leads that can better control dormant bacterial cells. If the compound is a substrate of efflux (more effective against persister cells), it may be applied with other antibiotics together to synergistically target both normal and dormant populations, e.g., synergy between ampicillin and eravacycline/minocycline found in this study.

Another important area for future development is target binding which includes both target selection and binding affinity. A strong binding with the target is required to create a sufficient window for killing before the drug diffuses out or being extruded during wake-up. Although further work is needed to determine if there is a threshold binding strength, the results of minocycline indicate that a dissociation constant of 10^-7^M or less would have good potential. Another factor to consider is the availability of binding target. Although persister cells do not have genetic mutations, tolerance may induce phenotypic changes that reduce the availability of drug target. For example, the formation of persister cells is accompanied by suppression of protein production and thus a lower amount of key ribosomal proteins compared to normal cells. Furthermore, persister cells also contain inactive ribosomes (inactive 70S, 90S, and 100S ribosomes) as a means of preservation during stress [76,77]. Our findings showed that *E*. *coli* persister cells accumulated 2.6 times of minocycline compared to normal cells. However, since persister cells only contain about 25% of normal ribosomes compared to the normal cells [78,79], the ratio of intracellular minocycline molecules to the amount of target is probably more than 10 times higher. This helps explain the killing of persister cells since activation of ribosome complexes is a crucial step for persister resuscitation [80]. Future studies are needed to identify drug candidates with strong target binding in both normal and persister cells to eradicate both populations.

Besides persister cells, bacteria are known to enter other physiological states that are difficult to treat, one is the formation of nongrowing but metabolically active (NGMA) cells, also known as viable but nonculturable (VBNC) cells [81]. Because these cells are less active than normal cells, it is possible that the strategy reported here can also be effective given appropriate agent and treatment condition. It is documented that these dormant cells require special environment and more time to resuscitate, which may require even stronger target binding (e.g. covalent bonding) to ensure the drug does not diffuse out before killing can occur. On the other hand, studies have shown that NGMAs have fewer ribosomes than the persister population [81]; therefore, a lower drug concentration might be effective to kill NGMAs. Further study will provide more insights into the potential of this strategy and the guidance for discovering better control agents.

Overall, the findings from this study demonstrate the feasibility to kill persister cells by antibiotics that can penetrate membranes through energy independent pathways (without active uptake) and have strong binding with the target. These agents can cause persister killing during “wake-up” when the extracellular stressors are removed. Developing more effective agents based on this strategy requires a better understanding of the structural effects of antimicrobials on persister killing and a capability to predict membrane penetration of different compounds. Identifying appropriate wake-up conditions is also important for further development of persister control strategies. Besides *E*. *coli* HM22 and an UPEC strain described above, we also validated this strategy against *P. aeruginosa* (to be published elsewhere). To further evaluate the potential of this new strategy, it is important to study this mechanism on other pathogenic species as well and if additional criteria needed added/tailored for different species, e.g. Gram-negative vs. Gram-positive bacteria and mycobacteria. It is also important to evaluate this strategy *in vivo*. This is part of our ongoing work.

## Methods

### Bacterial strains and growth media

*Escherichia coli* Top10, *E*. *coli* HM22 (AT984 *dapA* zde-264::Tn10 *hipA7*), *E*. *coli* BW25113, *E*. *coli* BW25113 *ΔacrB* [82], *E*. *coli* BW25113 *ΔacrA* [82], *E*. *coli* BW25113 *ΔtolC* [82], *S*. *aureus* ALC2085 [83], *Bacillus subtilis* 168 [84], and uropathogenic *E*. *coli* (UPEC) ATCC53505 were routinely cultured in Lysogeny broth (LB) containing 10g/L NaCl, 5g/L yeast extract, and 10 g/L tryptone. *E*. *coli* Top10/pRJW1 cultures were supplemented with 100 μg/mL of ampicillin to maintain the plasmid and 0.2% arabinose to induce *hipA* expression. *E*. *coli* HM22 cultures were supplemented with 25 μg/mL diaminopimelic acid (DPA) [15] to ensure its ability to make new cell wall proteoglycan. The cultures of *E*. *coli* MG1655 *tolC* reporter [61] were supplemented with 25 μg/mL of kanamycin.

### Persister isolation and treatment

Overnight cultures of *E*. *coli* HM22 were sub-cultured in LB supplemented with 25 μg/mL DPA with a starting OD_600_ of 0.05 until OD_600_ reached 0.3–0.45. The mid-exponential phase cultures were collected by centrifugation at 13,000 rpm for 3 min at room temperature. The amount of cells used for each treatment was adjusted to OD_600_ of 0.5 in 500 μL. They were then washed with phosphate buffered saline (PBS) (pH 7.4) three times. For the normal population, the cells were replenished with PBS and immediately treated with minocycline (Sigma Aldrich, St. Louis, MO, USA) for 1 h at 37°C with shaking at 200 rpm. After 1 h, the treated samples were collected by centrifugation and washed once with PBS to remove the remaining free antibiotic in the solution. The cells were then resuspended in PBS and plated on LB agar plates containing 25 μg/mL DPA to count CFU using the drop plate method [85]. To isolate persister cells, the cells in mid-exponential phase culture were treated with 100 μg/mL ampicillin for 3.5 h at 37°C with shaking at 200 rpm which resulted in ~1% of persister cells [15,39]. After isolation, the cells were washed three times with PBS to remove extracellular antibiotic and then proceeded to minocycline treatment as described above with a starting density of ~10^6^ cells [15,39]. Relative viability was normalized by the untreated population. Each experimental condition was tested with five biological repeats. It is important to note that minocycline is both pH sensitive and light sensitive. These factors were considered while performing the assay. The minocycline treatments of *E*. *coli* BW25113, and its mutants of *acrB*, *acrA*, *and tolC* were conducted in the same way as described above for the normal population. The tests for eravacycline, tetracycline, chloramphenicol, and rifamycin SV were carried out in the same way as minocycline.

### Checkerboard assay

Overnight cultures of *E*. *coli* HM22 were sub-cultured in LB supplemented with 25 μg/mL DPA with a starting OD_600_ of 0.05 until OD_600_ reached 0.3–0.45. The mid-exponential phase cultures were collected by centrifugation at 13,000 rpm for 3 min at room temperature. The amount of cells used for each treatment was adjusted to OD_600_ of 0.5 in 500 μL LB medium. Different concentrations of ampicillin was added and the samples were incubated for 3.5 h at 37°C with shaking at 200 rpm. After treatment, cells were washed three times with PBS and then treated with different concentrations of minocycline for 1 h in PBS at 37°C with shaking at 200 rpm. The treated samples were collected by centrifugation and washed once with PBS to remove the remaining free antibiotic in the solution. The cells were then resuspended in PBS and plated on LB agar plates containing 25 μg/mL DPA to count CFU using the drop plate method [85].

### Quantification of intracellular concentrations of minocycline, rifamycin SV, and eravacycline

The killing results of the reporter strain treated with *E*. *coli* lysate spiked with known concentrations of an antibiotic were used to generate a standard curve (S1 and S2 Figs) first (*E*. *coli* BW25113 *ΔtolC* for tetracycline, *B*. *subtilis* 168 for minocycline and rifamycin SV, and *S*. *aureus* for eravacycline), which was then used to determine the concentration in unknown samples. For both populations, the lysates from treated *E*. *coli* HM22 cells and untreated controls were collected and dried overnight, and then dissolved in PBS to treat the reporter strain. Cell lysates were extracted using chloroform after treatment as described above. Then cell debris was removed by centrifugation at 5,000 g for 5 min and the solvent was evaporated overnight in a vacuum desiccator. The samples were concentrated by 5 times before further analysis. To conduct LC-MS analysis, after overnight evaporation as described above, samples were resuspended in 50 μL of DI water. Antibiotics were quantified using a Thermo LTQ Orbitrap mass spectrometer at SUNY Upstate Medical University. A reporter strain-based bioassay was used to corroborate the results. Briefly, lysates extracted with chloroform from both treated and untreated samples were evaporated overnight in a vacuum desiccator after 5× concentration. The evaporated samples were dissolved in 100 μL sterile PBS (pH 7.4) with constant shaking for 5 min using a vortex mixer. The samples were then used to treat the reporter strain with an OD_600_ of 0.5 in 500 μL of PBS. After 1 h of incubation, the treated samples were collected by centrifugation and washed three times with PBS to remove the remaining free antibiotic. The cells were then resuspended with PBS and plated on LB agar plates to count CFU using the drop plate method. Antibiotic concentration was calculated based on the standard curve (S1 and S2 Figs). Individual cell volume of *E*. *coli* HM22 normal and *E*. *coli* HM22 persister were calculated based on microscopic images. Total cell numbers were obtained using a hemocytometer for each population.

### Validation of chloroform extraction

To validate if chloroform is effective in extracting the antibiotics after cell lysis, we performed a validation test. Briefly, 100 μL of antibiotic solution was mixed with 100 μL chloroform in a microcentrifuge tube by vortexing. Then the solution was centrifuged for 5 min at 5,000×g. After centrifugation, two distinct phases were seen with the aqueous phase on the top and the chloroform phase at the bottom. Each phase was collected separately and evaporated overnight in a desiccator. On the following day, the evaporated samples were resuspended in 100 μL PBS to dissolve antibiotic with constant shaking for 5 min using a vortex mixer. The samples were then transferred to a 96-well plate where absorbance readings were measured using an Epoch 2 Microplate Spectrophotometer (BioTek, Winooski, VT, USA). Readings for minocycline and eravacycline were taken at 360 nm and 370 nm, respectively. The concentrations were then calculated by comparing with a standard curve of absorbance with known concentrations of corresponding antibiotic. The partition coefficient was calculated based on the concentration extracted from the chloroform phase over the concentration extracted from the aqueous phase (S1 Table). Since chloroform was added to the sample as 5:1 (v/v chloroform: aqueous phase), we estimate that 93.7% of the antibiotic was extracted.

### Construction of pRJW1 carrying *P*_*BAD*_*-hipA*

The *hipA* gene was PCR amplified from *E*. *coli* DH5α with added restriction sites of *Nco*I and *EcoR*I, included in the forward and reverse primer sequences, respectively. The PCR product was then digested by *Nco*I and *EcoR*I and ligated into a similarly digested pBAD/HisD cloning vector to generate pRJW1. The plasmid was then transformed into *E*. *coli* Top10 by electroporation.

### Efflux activity

The results of membrane potential based on JC-1 staining was corroborated by monitoring efflux activities. To induce persister formation, overnight culture of *E*. *coli* Top10/pRJW1was sub-cultured with a starting cell density of 0.01 at OD_600_ and incubated till OD_600_ reached 0.15–0.2. This mid-exponential phase culture was supplemented with 0.2% arabinose and incubated for another 3 h at 37°C with shaking at 200 rpm to induce persister formation through the induction of *hipA* gene under the *P*_*BAD*_ promoter. Induced and uninduced *E*. *coli* Top10/pRJW1 cells in exponential cultures were washed and resuspended in PBS as described above. Both samples were stained with 20 μg/mL ethidium bromide (EtBr) [86] and analyzed after 0, 5, 10, and 30 min of incubation to compare the efflux of EtBr. Briefly, excess extracellular EtBr was gently washed away with PBS after staining and 200 μL cell suspension of each sample was transferred to a clear bottomed black walled 96 well plate to measure the signal from EtBr-nucleic acid complex formed in the cells using a microplate spectrophotometer (Model FLx800 microplate reader, Bio-Tek Instruments, Winooski, VT, USA). The JC-1 signal was measured in PBS with excitation at 360 nm and emission at 590 nm.

Meanwhile, a portion of cells from each induced or uninduced population was taken to determine the number of persister cells. These samples were treated with 5 μg/mL ofloxacin for 3 h at 37°C with shaking at 200 rpm to kill normal cells as described previously [87]. The persister cells harvested by centrifugation were washed with PBS three times to remove any remaining antibiotic in the medium. Then the cells were re-suspended in PBS and plated on LB agar plates to count CFU using the drop plate method as described previously [88]. Each experimental condition was tested with three biological replicates.

### Flow cytometry

Flow cytometry analysis was used to corroborate the EtBr efflux results of *E*. *coli* Top10/pRJW1. Wild-type *E*. *coli* K12 and its efflux pump mutant *E*. *coli* Δ*acrB* were used as positive (low EtBr signal) and negative (max EtBr signal) controls, respectively. The exponential cultures of induced and uninduced *E*. *coli* Top10/pRJW1 were stained as described above and the fluorescence signal intensity of each cell in the population was determined using an Accuri C6 flow cytometer (BD Biosciences, San Jose, CA, USA).

### Characterizing membrane potential

The membrane potentials of normal (uninduced) and persister (induced) cells of *E*. *coli* Top10/pRJW1 were compared using JC-1 potentiometric dye, which is commonly used to stain mitochondria of eukaryotic cells [89] and bacterial membranes [90,91] based on its membrane potential-induced aggregation (red fluorescence). JC-1 also diffuses into the cytoplasm and emits green fluorescence irrespective of the metabolic stage of a cell; thus, the red/green ratio of JC-1 staining is positively correlated with membrane potential [89]. To induce persister formation, overnight culture of *E*. *coli* Top10/pRJW1was sub-cultured with a starting cell density of 0.01 at OD_600_ (optical density at 600 nm) and incubated till OD_600_ reached 0.15–0.2. This mid-exponential phase culture was supplemented with 0.2% arabinose and incubated for another 3 h at 37°C with shaking at 200 rpm to induce persister formation through the induction of *hipA* gene under the *P*_*BAD*_ promoter. After 3 h of incubation, 50 μg/mL tetracycline was added and the culture was incubated for another 0.5 h to further induce persister formation by inhibiting protein synthesis as reported previously [60]. Then the cells were collected by centrifuging at 10,000×g for 8 min and washed twice with PBS. Ten μL JC-1 dye was added in each 300 μL cell sample and mixed by gentle pipetting. The samples were incubated at 37°C for 15 min in dark. After incubation, excess JC-1 dye was removed by washing with PBS. Then samples were analyzed with flow cytometry to compare membrane potentials by characterizing populations based on red and green fluorescence. Cells emitting high red/green fluorescence ratios were identified as cells with high membrane potential, and vice versa.

### Minocycline depotentiation activity

An overnight culture of *E*. *coli* HM22 was sub-cultured in LB medium supplemented with DPA with a starting OD_600_ of 0.05 until OD_600_ reached 0.3–0.45. The mid-exponential culture was collected by centrifugation at a speed of 13,000 rpm for 3 min at room temperature. The amount of cells used for each treatment was adjusted to an OD_600_ of 0.5 in 500 μL LB. The cells were then washed three times with PBS (pH 7.4), and pretreated with 100 μM of CCCP (Sigma Aldrich; dissolved in dimethyl sulfoxide) at 37°C for 10 min in PBS, followed by immediate treatment with 100 μg/mL of minocycline at 37°C for 1 h. Then the treated samples were collected by centrifugation and washed once with PBS to remove the remaining free antibiotic. The cells were resuspended in PBS and plated on LB agar plates containing 25 μg/mL DPA to count CFU using the drop plate method. Each experimental condition was tested with three biological replicates.

### Microscopy and image analysis

Treated and untreated samples were washed once with PBS (pH 7.4). Cells were then immediately labeled with LIVE/DEAD BacLight bacterial viability kit (Life Technologies Inc., Carlsbad, CA) with a final concentration of 7.5 μM SYTO9 and 30 μM propidium iodide. After 15 min of staining, the cells were pelleted to remove the staining solution, re-suspended in PBS and vortexed briefly. Labeled cells were then imaged on microscope slides using an Axio Imager M1 fluorescence microscope (Carl Zeiss Inc., Berlin, Germany) with an Orca-Flash 4.0 LT camera (Hamamatsu Photonics, Hamamatsu City, Japan). At least 5 random spots were imaged for each sample. The mean gray value intensity was used to calculate the mean intensity generated from each channel (green and red). Each condition was tested with three biological replicates and 5 images were randomly taken from each sample.

### Antibiotic diffusion assay

Mid-exponential cultures of *E*. *coli* HM22 were collected by centrifugation at 13,000 rpm for 3 min at room temperature. The cells were resuspended with LB after washing and then 100 μg/mL ampicillin was added. The samples were incubated for 3.5 h at 37°C with shaking at 200 rpm to isolate persister cells as described previously [15]. After isolation, the cells were washed three times with PBS to remove the antibiotic and then proceeded to minocycline or eravacycline treatment for 1 h at 37°C with shaking at 200 rpm. Untreated cells were incubated in the absence of minocycline or eravacycline for 1 h at 37°C with shaking at 200 rpm. At each designated time point, 1 mL of the cell culture was collected, washed with PBS, and centrifuged at a speed of 13,000 rpm for 3 min. Treated persister cells and untreated controls were washed and resuspended with LB medium supplemented with DPA, and incubated at 37°C with shaking at 200 rpm. At each designated time point, samples were collected to quantify intracellular antibiotic concentration as described above and to determine growth by measuring OD_600_.

### TolC activity during persister wake-up

An overnight culture of *tolC* reporter (*E*. *coli* MG1655 with *tolC* promoter-*gfp* fusion) was sub-cultured in LB medium supplemented with 25 μg/mL kanamycin with a starting OD_600_ of 0.05 until OD_600_ reached 0.3–0.45. Persister cells were isolated by ampicillin treatment as described above. Both normal and persister cells were washed and resuspended in 200 μL LB supplemented with 25 μg/mL kanamycin and transferred to a 96 well plate. The samples were incubated at 37°C with shaking at 200 rpm. At different timepoints, the cell density (OD_600_) and fluorescence (485nm/535nm) were quantified using a Synergy 2 Multi-Mode Microplate Reader (Bio-Tek Instruments, Winooski, VT, USA). In addition, samples were imaged using an Axio Imager M1 fluorescence microscope (Carl Zeiss Inc., Berlin, Germany) with an Orca-Flash 4.0 LT camera (Hamamatsu Photonics, Hamamatsu City, Japan).

### Molecular dynamic simulations

All-atom MD simulations were performed using GROMACS molecular dynamics package (version 2016.4) [66,92] and CHARMM36 force field [93] and TIP4P for water[94]. The force field for eravacycline was generated using an Automated Force Field Topology Builder (ATB) and Repository [95]. Using the 30 ribosomal subunit molecular structure (PDB 4YHH), we docked minocycline and eravacycline into the binding pocket. The system was solvated with explicit water and neutralized to balance the charge. Energy minimization was performed using the steepest-decent algorithm [96] until the maximum force on any bead was below the tolerance parameter of 100 kJmol^−1^nm^−1^. Periodic boundary conditions were applied in all three dimensions. Equilibration runs were performed in isothermal-isochoric (NVT) and isothermal-isobaric (NPT) ensembles equilibration runs were performed for 0.1 ns and 0.05 ns, respectively. The long range electrostatic interactions are computed using the Particle Mesh Ewald electrostatics [97]. The systems were simulated at 1 bar pressure using isotropic Parrinello-Rahman barostat [98] with a coupling constant τp = 2.0 ps and compressibility factor of 4.5×10^−5^ bar^−1^. The temperature was maintained at 298 K by independently coupling the water, nucleotides, and protein molecules to an external velocity rescaling thermostat [99] with τT = 0.1 ps. The neighbor list was updated every 5 steps using 1.0 nm for short-range van der Waals and electrostatic cutoffs. Bonds with H-atoms were constrained with the LINCS algorithm [100]. The production NPT simulations were performed for 1 μs for all the systems and post simulation analyses were performed using in-built GROMACS utilities. Molecular visualization and graphics were generated using visual molecular dynamics (VMD) software [101].

### PDMS surface preparation

Biofilms were grown on PDMS (polydimethylsiloxane) surfaces. Using the Sylgard® 184 elastomer kit, base and curing agent at 10:1 (w/w) ratio were poured, mixed, and vacuumed in a 50 mL falcon tube. The PDMS was polymerized at 60°C for 24 h after pouring onto a sterile 25 cm petri dish. After 24 h, rectangular pieces of PDMS surfaces (10 mm by 5 mm, 2 mm thick) were cut out using a sterile razor. The PDMS surfaces were then transferred into new petri dish and sterilized under UV for 1 h.

### Biofilm assay

Biofilms were grown on PDMS surfaces. Briefly, an overnight culture of UPEC (~16 h) was used to inoculate LB medium in a petri dish containing PDMS surfaces with a starting OD_600_ of 0.05. The biofilm culture was grown for 48 h at 37°C. After that, each PDMS surface with biofilm was washed three times with PBS, transferred into a 10 cm petri dish, and treated for 1 h at 37°C in PBS with or without eravacycline. After treatment, the tubes containing biofilm samples were gently sonicated for 1 min and vortexed for 30 s to detach biofilm cells from the PDMS surface. The number of viable biofilm cells were determined by counting CFU.

### Statistical analysis

Error bars in all figures represent standard error of the mean. All data were analyzed using one-way ANOVA or two-way ANOVA followed by Tukey test if not noted otherwise using SAS version 9.13 (SAS Institute, Cary, NC, USA). Differences with *p*<0.05 were considered to be statistically significant (* *p*-value≤ 0.05, ** *p*-value ≤ 0.01, *** *p*-value ≤ 0.001 and ****p-value ≤ 0.0001).

## Supporting information

S1 Table Validation of chloroform extraction. The partition coefficient of minocycline and eravacycline was calculated based on the concentration in extracted chloroform phase over the concentration in aqueous phase.
(TIFF)

S1 Fig Overview of the reporter strain-based assay to quantify intracellular concentration of antibiotics. (A) Schematic overview of the lysate collection after normal and persister cells of *E*. *coli* HM22 were treated with minocycline. (B-C) The reporter strain *B*. *Subtilis* 168 was used to evaluate the killing activities of cell lysates (B) and establish a standard curve (C) for quantification of antibiotic concentration in unknown samples. (D) The concentration obtained from the standard curve and killing activity was normalized by the number of cell and cell volume.
(TIF)

S2 Fig Standard curves of tetracycline, rifamycin SV, and eravacycline. (A) The standard curve of tetracycline was generated using the reporter strain *E*. *coli ΔtolC* treated with *E*. *coli* HM22 lysates supplemented with known concentrations of tetracycline. (B) The standard curve of rifamycin SV was generated using the reporter strain *B*. *subtilis* 168 treated with *E*. *coli* HM22 lysates supplemented with known concentrations of rifamycin SV. (C) The standard curve of eravacycline was generated using the reporter strain *S*. *aureus* ALC2085 treated with *E*. *coli* HM22 lysates supplemented with known concentrations of eravacycline.
(TIF)

S3 Fig Efflux activity during persister wake-up. *E*. *coli* MG1655 *tolC* promoter *-gfp* fusion strain was used as reporter to monitor the expression of *tolC*, which is part of the AcrAB-TolC efflux pump. Persister cells and exponential phase cells were monitored after being transferred to LB medium to wake up. At different timepoints, GFP signal (A) and cell growth based on OD_600_ (B) were measured using a plate reader.
(TIFF)

S4 Fig Inactivation of efflux pumps sensitized normal cells to eravacycline. The graph shows the CFUs after eravacycline treatment of *E*. *coli* BW25113, *E*. *coli* BW25113 *ΔacrA*, *E*. *coli* BW25113 *ΔtolC*, and *E*. *coli* BW25113 *ΔacrB*. Means ± SE are shown (n = 3).
(TIF)

S5 Fig Viability of UPEC normal, persister and biofilm cells after treatment with eravacycline. (A) Effects on the viability of planktonic normal (black bars) and persister (patterned bars) cells of UPEC. Means ± SE are shown (n>3). (B) Effects on 48-h UPEC biofilms. The 48-h biofilms were treated with different concentrations of eravacycline for one hour. Means ± SE are shown (n = 3). Biofilms were cultured on polydimethylsiloxane (PDMS; a polymer commonly used for manufacturing urinary catheters). Means ± SE are shown (n = 3).
(TIF)
