# Supplementary figures and images for: Persister control by leveraging dormancy associated reduction of antibiotic efflux
Source: PLoS Pathog. 2021 Dec 10;17(12):e1010144. doi: 10.1371/journal.ppat.1010144 (PMC8716142; doi:10.1371/journal.ppat.1010144)

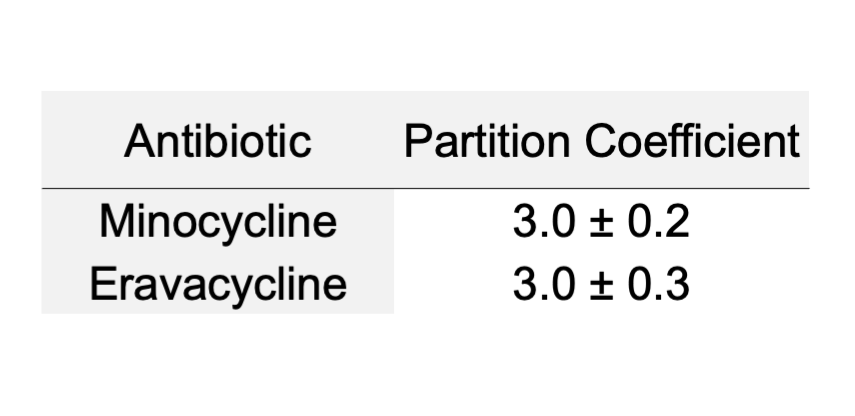

Supplement: S1 Table — The partition coefficient of minocycline and eravacycline was calculated based on the concentration in extracted chloroform phase over the concentration in aqueous phase. (TIFF) [file ppat.1010144.s001.tiff]

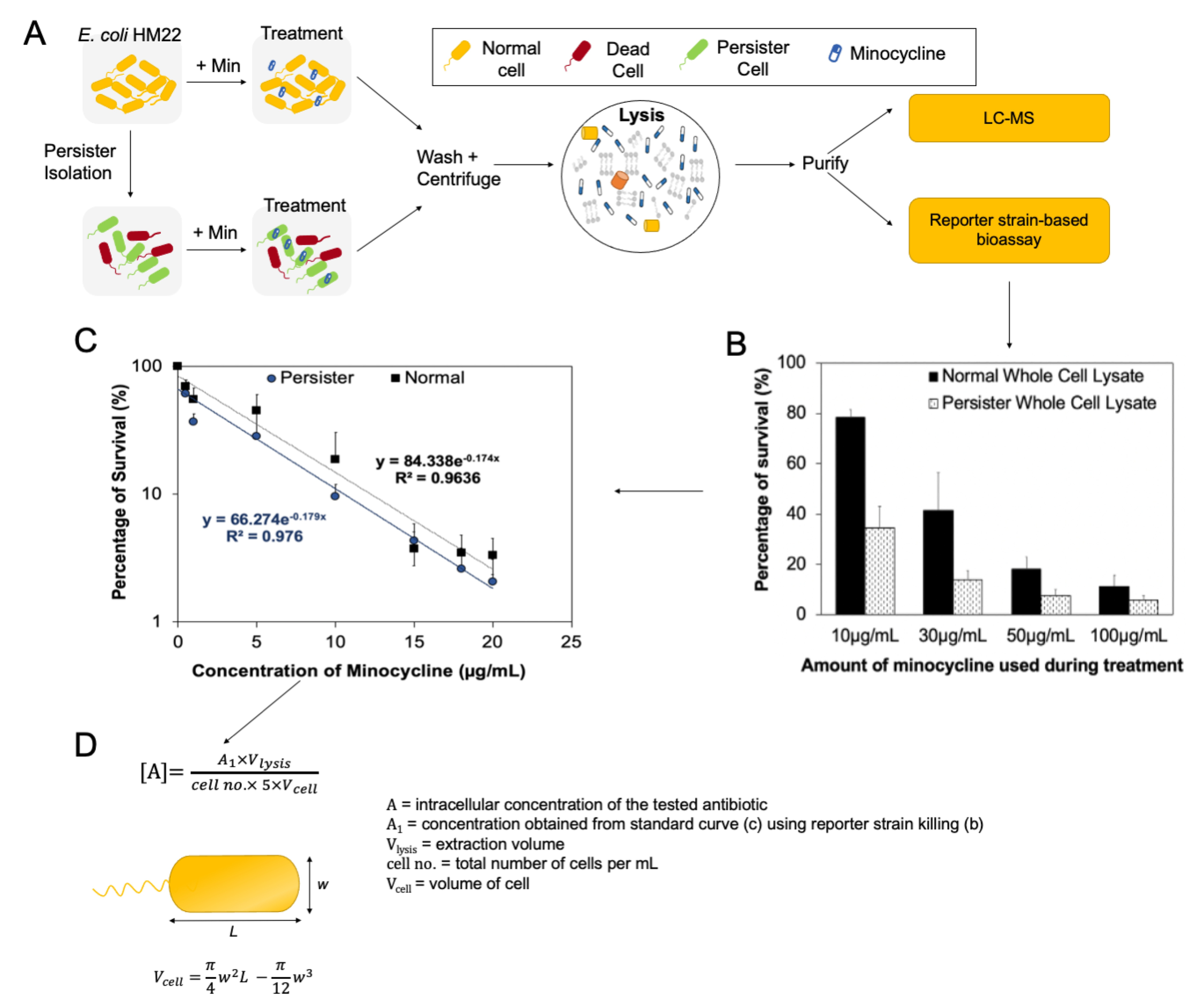

Supplement: S1 Fig — (A) Schematic overview of the lysate collection after normal and persister cells of E. coli HM22 were treated with minocycline. (B-C) The reporter strain B. Subtilis 168 was used to evaluate the killing activities of cell lysates (B) and establish a standard curve (C) for quantification of antibiotic concentration in unknown samples. (D) The concentration obtained from the standard curve and killing activity was normalized by the number of cell and cell volume. (TIF) [file ppat.1010144.s002.tif]

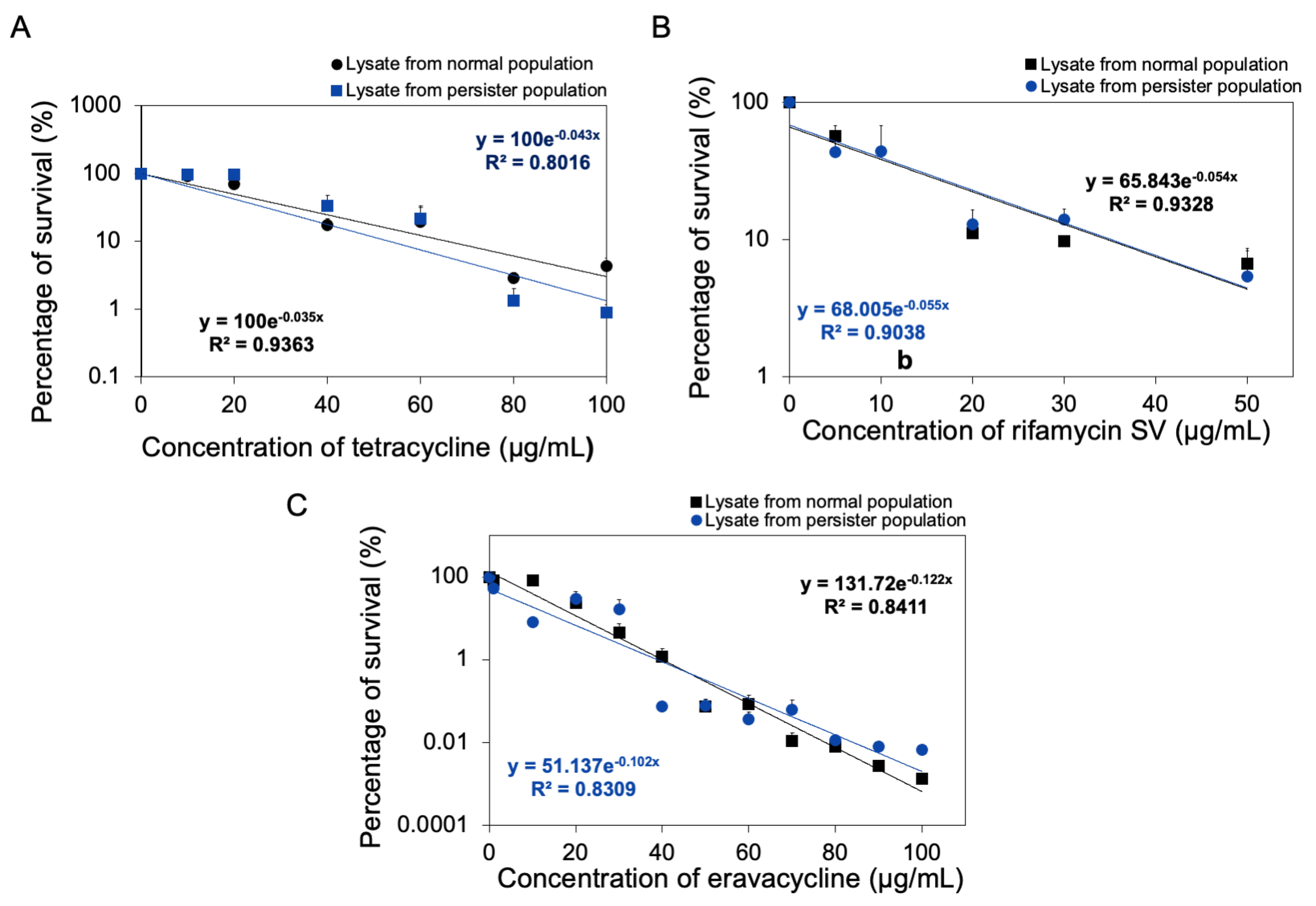

Supplement: S2 Fig — (A) The standard curve of tetracycline was generated using the reporter strain E. coli ΔtolC treated with E. coli HM22 lysates supplemented with known concentrations of tetracycline. (B) The standard curve of rifamycin SV was generated using the reporter strain B. subtilis 168 treated with E. coli HM22 lysates supplemented with known concentrations of rifamycin SV. (C) The standard curve of eravacycline was generated using the reporter strain S. aureus ALC2085 treated with E. coli HM22 lysates supplemented with known concentrations of eravacycline. (TIF) [file ppat.1010144.s003.tif]

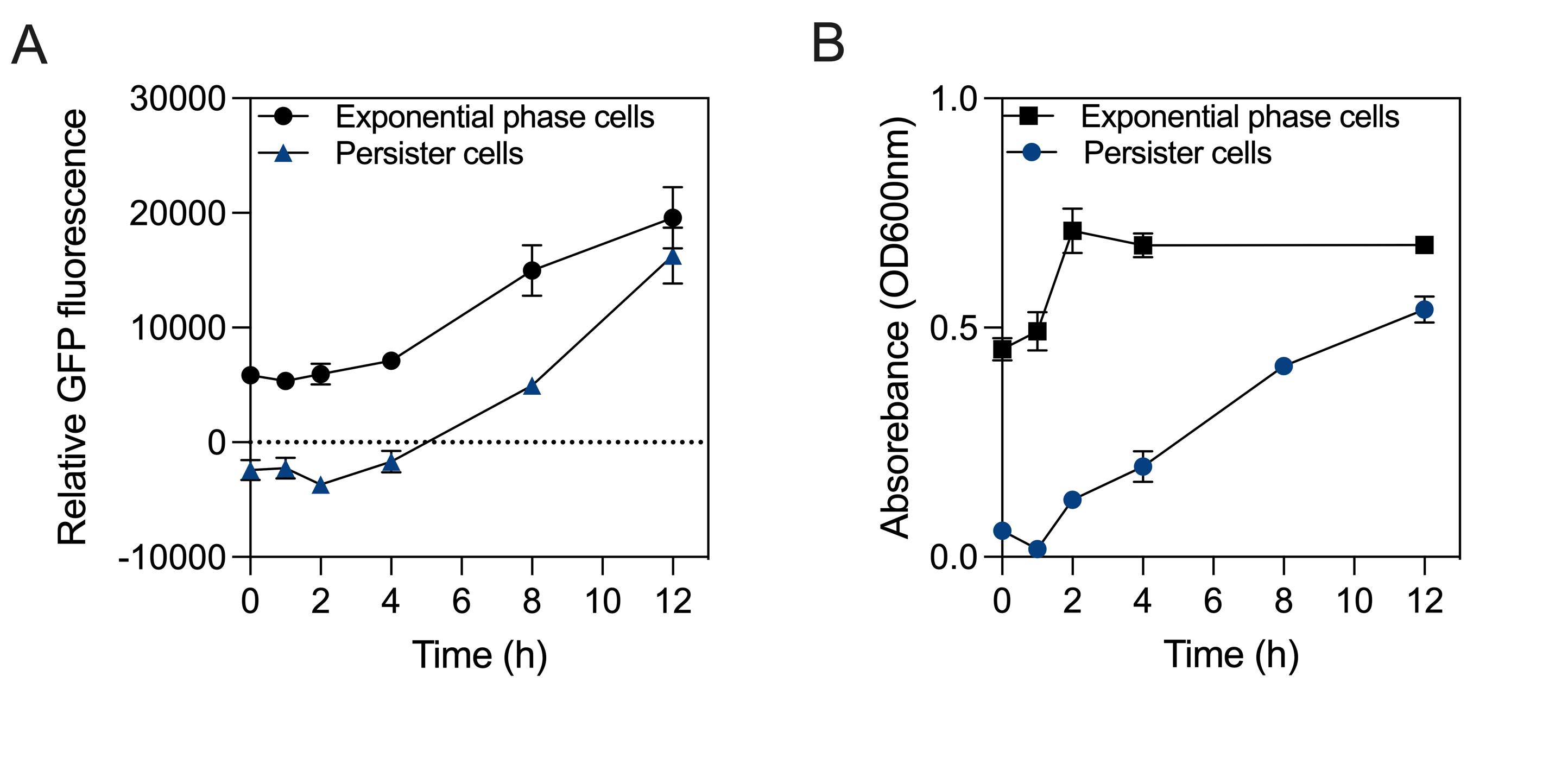

Supplement: S3 Fig — E. coli MG1655 tolC promoter -gfp fusion strain was used as reporter to monitor the expression of tolC, which is part of the AcrAB-TolC efflux pump. Persister cells and exponential phase cells were monitored after being transferred to LB medium to wake up. At different timepoints, GFP signal (A) and cell growth based on OD600 (B) were measured using a plate reader. (TIFF) [file ppat.1010144.s004.tiff]

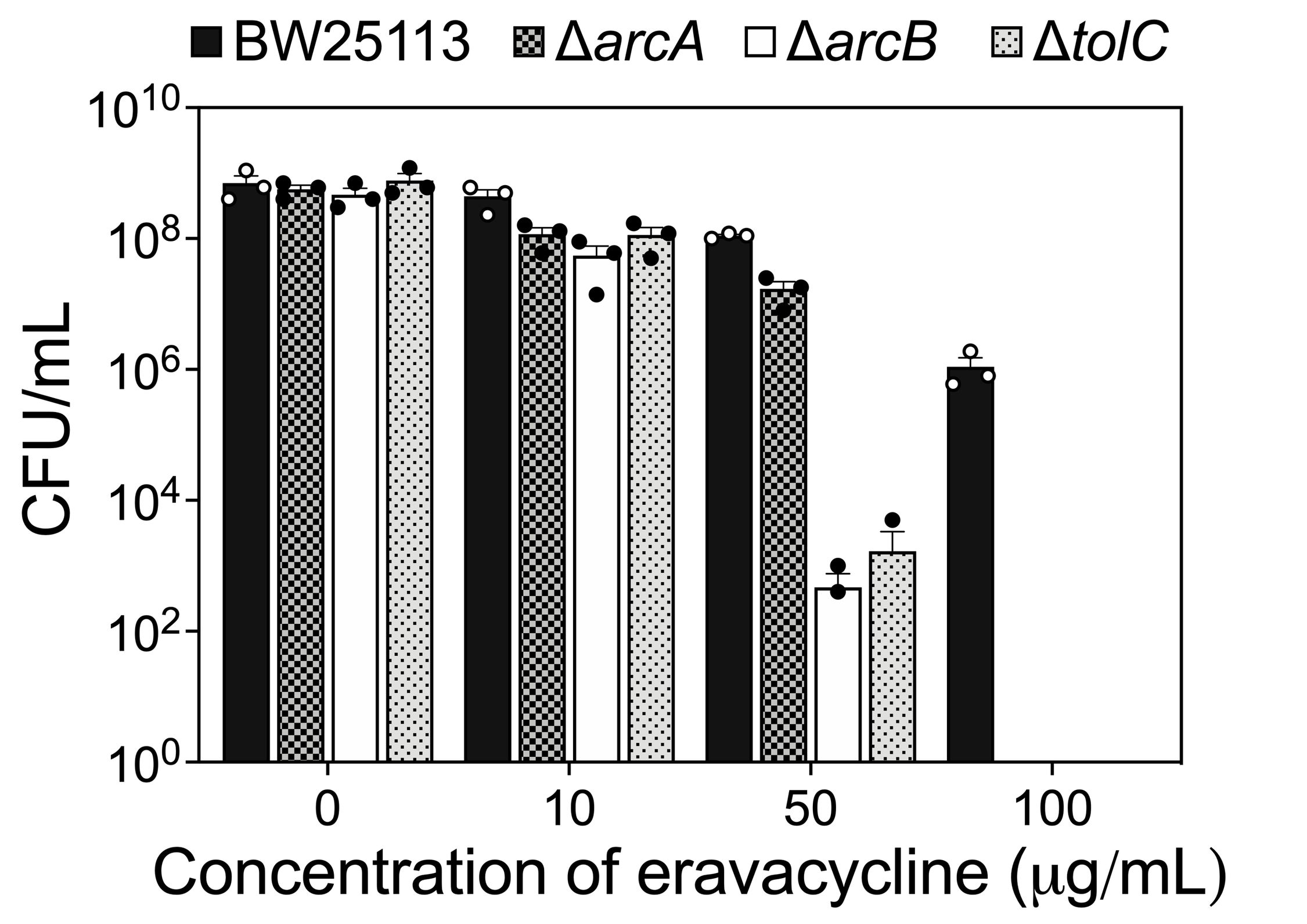

Supplement: S4 Fig — The graph shows the CFUs after eravacycline treatment of E. coli BW25113, E. coli BW25113 ΔacrA, E. coli BW25113 ΔtolC, and E. coli BW25113 ΔacrB. Means ± SE are shown (n = 3). (TIF) [file ppat.1010144.s005.tif]

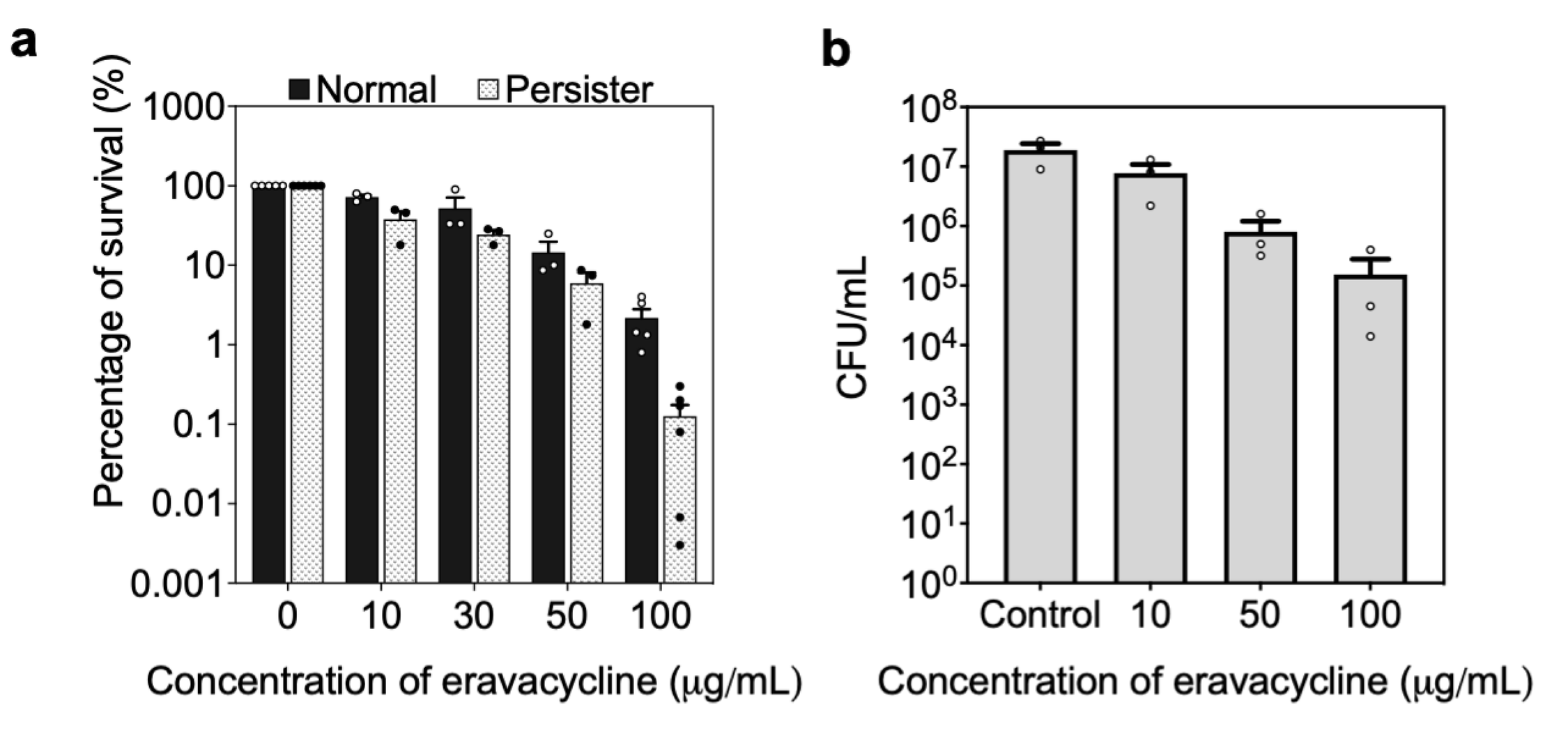

Supplement: S5 Fig — (A) Effects on the viability of planktonic normal (black bars) and persister (patterned bars) cells of UPEC. Means ± SE are shown (n>3). (B) Effects on 48-h UPEC biofilms. The 48-h biofilms were treated with different concentrations of eravacycline for one hour. Means ± SE are shown (n = 3). Biofilms were cultured on polydimethylsiloxane (PDMS; a polymer commonly used for manufacturing urinary catheters). Means ± SE are shown (n = 3). (TIF) [file ppat.1010144.s006.tif]
